# The flavonoid 7,8-DHF fosters prenatal brain proliferation potency in a mouse model of Down syndrome

**DOI:** 10.1038/s41598-021-85284-5

**Published:** 2021-03-18

**Authors:** Fiorenza Stagni, Beatrice Uguagliati, Marco Emili, Andrea Giacomini, Renata Bartesaghi, Sandra Guidi

**Affiliations:** 1 Department for Life Quality Studies, University of Bologna, Rimini, Italy; 2 Department of Biomedical and Neuromotor Sciences, Physiology Building, University of Bologna, Piazza di Porta San Donato 2, 40126 Bologna, BO Italy

**Keywords:** Development of the nervous system, Neurodevelopmental disorders

## Abstract

Neurogenesis impairment is a key determinant of intellectual disability in Down syndrome (DS), a genetic pathology due to triplication of chromosome 21. Since neurogenesis ceases after birth, apart in the hippocampus and olfactory bulb, the only means to tackle the problem of neurogenesis impairment in DS at its root is to intervene during gestation. A few studies in DS mouse models show that this is possible, although the drugs used may raise caveats in terms of safety. We previously found that neonatal treatment with 7,8-dihydroxyflavone (7,8-DHF), a flavonoid present in plants, restores hippocampal neurogenesis in the Ts65Dn model of DS. The goal of the current study was to establish whether prenatal treatment with 7,8-DHF improves/restores overall brain proliferation potency. Pregnant Ts65Dn females received 7,8-DHF from embryonic day 10 until delivery. On postnatal day 2 (P2) the pups were injected with BrdU and were killed after either 2 h or 52–60 days (P52–60). Evaluation of the number of proliferating (BrdU+) cells in various forebrain neurogenic niches of P2 mice showed that in treated Ts65Dn mice proliferation potency was improved or even restored in most of the examined regions, including the hippocampus. Quantification of the surviving BrdU+ cells in the dentate gyrus of P52–60 mice showed no difference between treated and untreated Ts65Dn mice. At P52–60, however, treated Ts65Dn mice exhibited a larger number of granule cells in comparison with their untreated counterparts, although their number did not reach that of euploid mice. Results show that 7,8-DHF has a widespread impact on prenatal proliferation potency in Ts65Dn mice and exerts mild long-term effects. It remains to be established whether treatment extending into the neonatal period can lead to an improvement in brain development that is retained in adulthood.

## Introduction

Triplication of chromosome 21 causes a constellation of developmental abnormalities known as Down syndrome (DS). While some developmental abnormalities are inconsistent in their occurrence, brain hypotrophy and intellectual disability are a constant feature of DS that prevents those affected from leading an autonomous life. It is now clear that the typically hypotrophic brain of infants with DS is due to a widespread reduction in the number of brain neurons. Evidence in fetuses with DS and mouse models of DS shows that this reduction is caused by an impairment of neurogenesis that is already detectable during very early gestation^1–6^. Most brain neurons are generated prenatally with the exception of those of the hippocampal dentate gyrus and olfactory bulb which continue to generate postnatally^7–10^. The former derive from the subgranular zone of the dentate gyrus and the latter derive from the subventricular zone of the lateral ventricle. Previous evidence in the Ts65Dn mouse, the most widely used model of DS, showed that pharmacological treatments in the neonatal period^11–16^ or during adulthood^17–21^ are able to correct neurogenesis alterations in the hippocampal dentate gyrus. This evidence is of obvious relevance because it shows that it is possible to pharmacologically correct the altered proliferation program of trisomic neural progenitors. However, considering the time course of neurogenesis, which is almost exclusively limited to the prenatal period, therapeutic interventions aimed at correcting brain development in DS should be started in utero. In a previous study we tackled the problem at its root and treated Ts65Dn mice during gestation with fluoxetine, an antidepressant that is known to increase neurogenesis^22^. We found that treatment rescued neurogenesis and cellularity in a number of brain regions, from the forebrain to the cerebellum. Importantly, these effects translated into a behavioral rescue when mice reached adulthood^22^. At this point, the scientific community is faced with two critical questions which are intermingled. (1) Are treatments that were effective in mouse embryos equally effective in fetuses with DS? (2) Can treatments explored in mouse models be ethically proposed during pregnancy? The second question is of great importance because pharmacological treatments that correct neurogenesis impairment should also be free of side effects, in particular during the extremely delicate fetal period. Choline is one of the treatments that are effective in a model of DS^23–25^, and has been proven to be safe when tested in a control trial in pregnant women^26^. Although the prenatal treatments attempted so far in mouse models appear to be free of adverse effects in mice (see^27^)^28^, their use in human beings may be a source of concern. For instance, although fluoxetine is a widely used antidepressant that may also be prescribed in children, its use during pregnancy may cause alterations in heart development^29^. Thus, identification of new treatments that are effective and putatively safe during gestation still remains a challenge. With this challenge in mind, we have been seeking to discover molecules that can rescue neurogenesis in DS but that, due to their chemical nature, may be reasonably proposed for human use during pregnancy. Flavonoids are natural compounds present in fruits and vegetables that have been used for centuries in traditional herbal medicine. Their use has beneficial effects in various organs, including the brain, where they appear to exert nootropic effects^30^. 7,8-dihydroxyflavone (7,8-DHF) is a natural flavonoid that binds with high affinity and specificity to the tropomyosin-related kinase B (TrkB) receptor for the brain derived neurotrophic factor (BDNF), activates its downstream signaling cascade^31^, penetrates the blood brain barrier^32^, promotes neurogenesis in the dentate gyrus^31^, increases dendritic spine density^33^, and exerts neurotrophic effects in various developmental disorders^34^. In a previous study we tested the effect of 7,8-DHF in the Ts65Dn mouse and found that early postnatal treatment with this flavonoid was able to restore hippocampal neurogenesis, dendritic spine density, and hippocampus-dependent memory, with no side effects on somatic development^14^. Based on these premises, in the current study we sought to establish whether embryonic treatment with 7,8-DHF positively affects prenatal proliferation potency. We have used here the same mouse model of DS and the same time window of prenatal treatment previously used for the treatment with fluoxetine^22^, in order to obtain comparative information regarding the efficacy of these two treatments.

## Results

### Widespread mitotic activity in the forebrain of P2 mice

**Figure 1:**
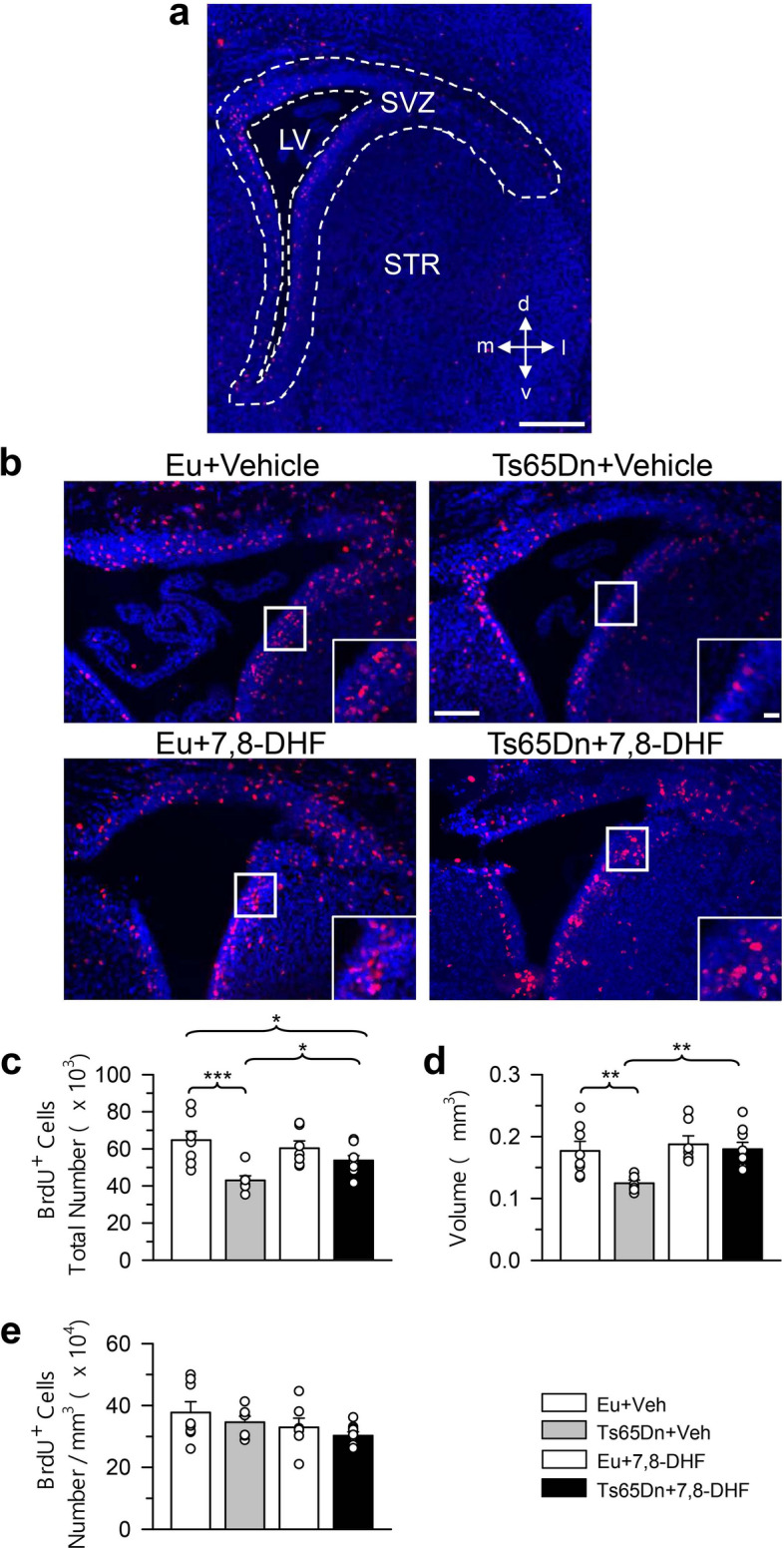
Effect of embryonic treatment with 7,8-DHF on neural precursor proliferation in the rostral subventricular zone of P2 Ts65Dn and euploid mice. (**a**) Section immunostained for BrdU and Hoechst from the rSVZ showing the region (area enclosed by the stippled lines) where BrdU-positive cells were counted. (**b**) Sections immunostained for BrdU and Hoechst of an animal from each experimental group. These animals received one injection of BrdU on P2 and were sacrificed after 2 h. Calibration bars: (**a**) = 200 μm; (**b**) = 100 μm; insert = 20 μm. The inserts in (**b**) show zoomed images of the boxed area with examples of individual BrdU-positive cells (red cells). (**c**–**e**) Total number of BrdU-positive cells in the rSVZ (**c**), volume of the rSVZ (**d**), and density of BrdU-positive cells in the rSVZ (**e**) of untreated euploid (n = 8) and Ts65Dn (n = 7) mice and euploid (n = 7) and Ts65Dn (n = 9) mice treated with 7,8-DHF. Values (mean ± SE) in (**c**–**e**) refer to one hemisphere. The scatterplots over each column represent the values of individual cases for each group. *p < 0.05; **p < 0.01; ***p < 0.001 (planned comparisons after one-way ANOVA). Images were acquired using NIS-Elements AR 4.30.02 (https://www.microscope.healthcare.nikon.com/it_EU/products/software/nis-elements/nis-elements-advanced-research). *Abbreviations 7,8-DHF* 7,8-dihydroxyflavone, *d* dorsal, *Eu* euploid, *l* lateral, *LV* lateral ventricle, *m* medial, *STR* striatum, *SVZ* subventricular zone, *v* ventral, *Veh* vehicle.

**Figure 2:**
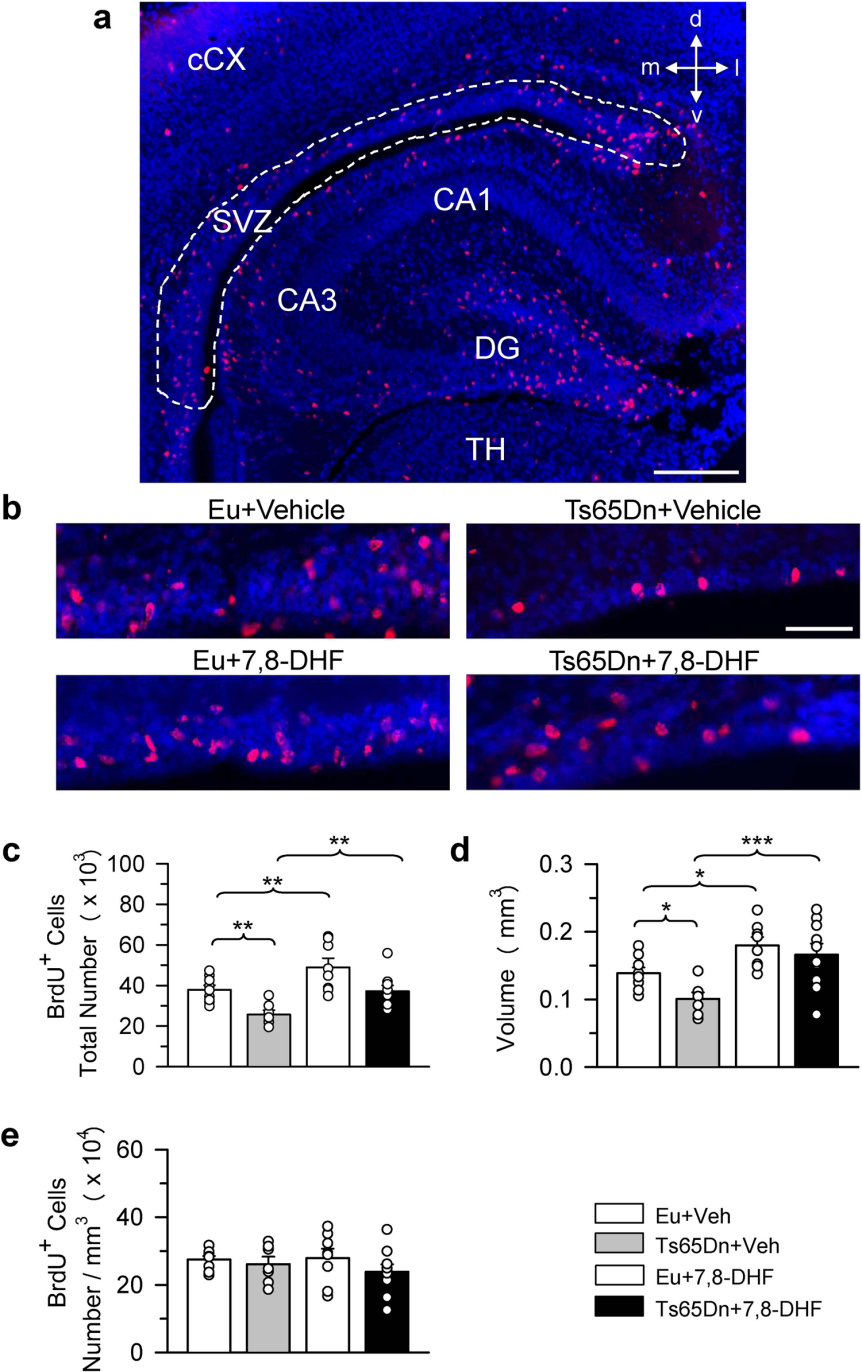
Effect of embryonic treatment with 7,8-DHF on neural precursor proliferation in the caudal subventricular zone of P2 Ts65Dn and euploid mice. (**a**) Section immunostained for BrdU and Hoechst from the cSVZ showing the region (area enclosed by the stippled line) where BrdU-positive cells were counted. (**b**) Sections immunostained for BrdU and Hoechst of an animal from each experimental group. These animals received one injection of BrdU on P2 and were sacrificed after 2 h. Calibration bars: (**a**) = 200 μm; (**b**) = 50 μm. (**c**–**e**) Total number of BrdU-positive cells in the cSVZ (**c**), volume of the cSVZ (**d**), and density of BrdU-positive cells in the cSVZ (**e**) of untreated euploid (n = 9) and Ts65Dn (n = 7) mice and euploid (n = 8) and Ts65Dn (n = 10) mice treated with 7,8-DHF. Values (mean ± SE) in (**c**–**e**) refer to one hemisphere. The scatterplots over each column represent the values of individual cases for each group. *p < 0.05; **p < 0.01; ***p < 0.001 (planned comparisons after one-way ANOVA). Images were acquired using NIS-Elements AR 4.30.02 (https://www.microscope.healthcare.nikon.com/it_EU/products/software/nis-elements/nis-elements-advanced-research). *Abbreviations 7,8-DHF* 7,8-dihydroxyflavone, *CA1, CA3* hippocampal fields, *cCX* caudal cortex, *d* dorsal, *DG* dentate gyrus, *Eu* euploid, *l* lateral, *m* medial, *SVZ* subventricular zone, *TH* thalamus, *v* ventral, *Veh* vehicle.

**Figure 3:**
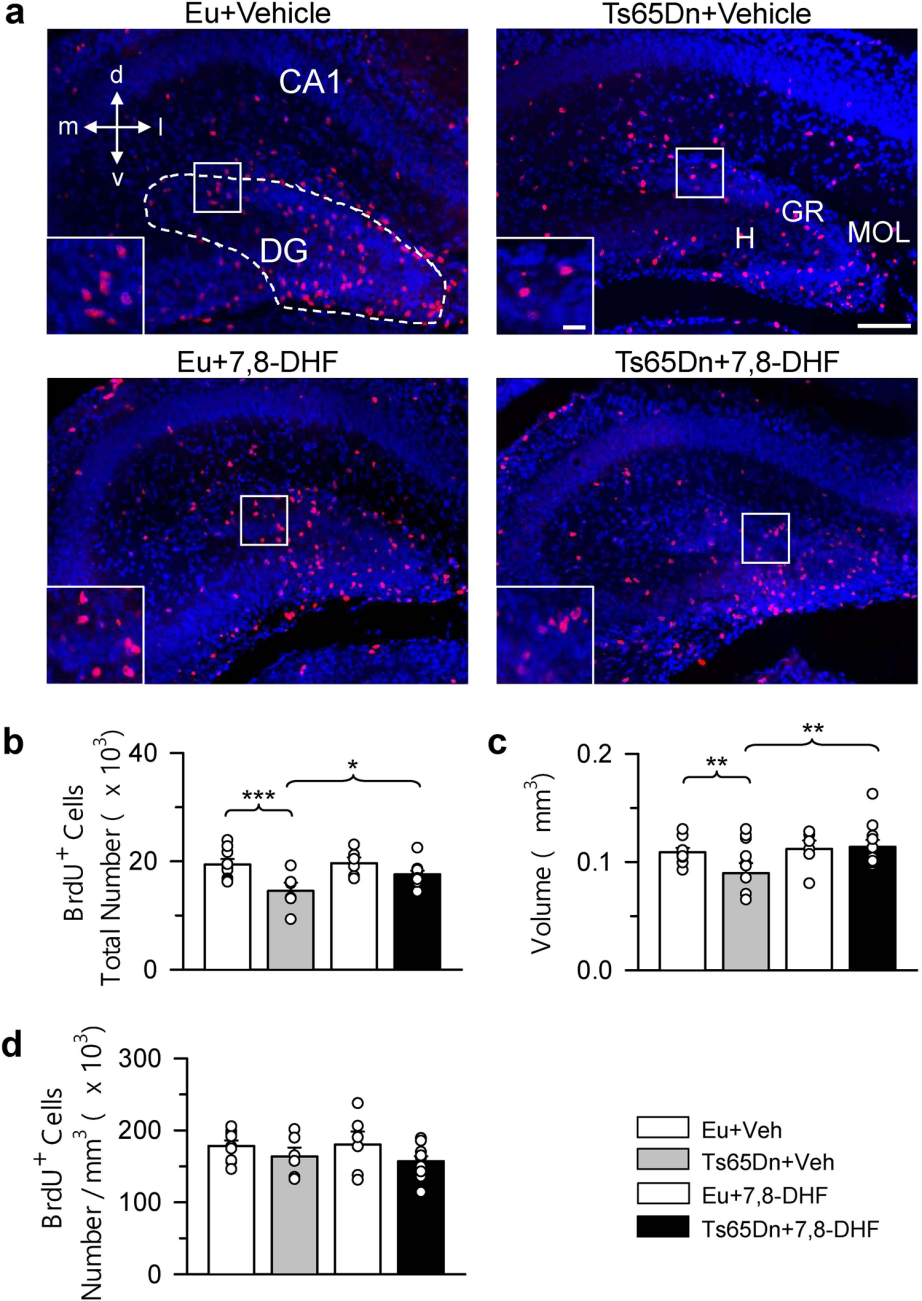
Effect of embryonic treatment with 7,8-DHF on neural precursor proliferation in the dentate gyrus of P2 Ts65Dn and euploid mice. (**a**) Examples of sections immunostained for BrdU and Hoechst from the DG of an animal from each experimental group. These animals received one injection of BrdU on P2 and were sacrificed after 2 h. Calibration = 100 μm. The inserts in (**a**) show zoomed images (calibration bar = 20 μm) of the boxed area with examples of individual BrdU-positive cells. (**b**–**d**) Total number of BrdU-positive cells in the hilus + subgranular zone + granule cell layer of the DG (**b**), volume of the DG (**c**), and density of BrdU-positive cells in the DG (**d**) of untreated euploid (n = 9) and Ts65Dn (n = 6; n = 5 for the volume and density) mice and euploid (n = 6) and Ts65Dn (n = 11; n = 10 for the volume and density) mice treated with 7,8-DHF. Values (mean ± SE) in (**b**–**d**) refer to one hemisphere. The scatterplots over each column represent the values of individual cases for each group. **p < 0.01; ***p < 0.001 (planned comparisons after one-way ANOVA). Images were acquired using NIS-Elements AR 4.30.02 (https://www.microscope.healthcare.nikon.com/it_EU/products/software/nis-elements/nis-elements-advanced-research). *7,8-DHF* 7,8-dihydroxyflavone, *CA1* hippocampal field, *d* dorsal, *DG* dentate gyrus, *Eu* euploid, *GR* granule cell layer, *H* hilus, *l* lateral, *m* medial, *MOL* molecular layer, *v* ventral, *Veh* vehicle.

**Figure 4:**
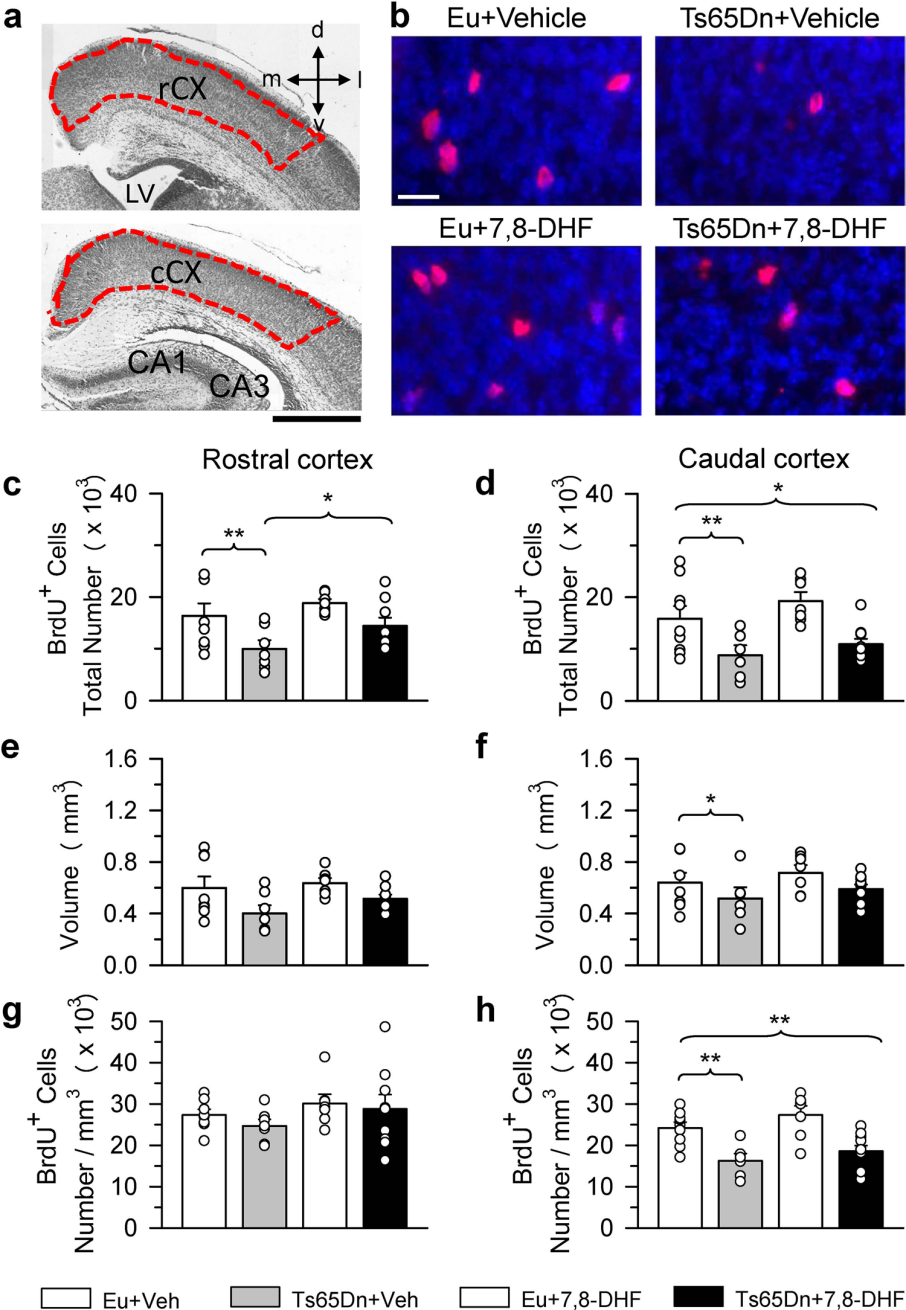
Effect of embryonic treatment with 7,8-DHF on proliferating cells in the neocortex of P2 Ts65Dn and euploid mice. (**a**) Nissl-stained sections across the rostral (upper panel) cortex and caudal (lower panel) cortex showing the regions (area enclosed by a stippled line) where BrdU-positive cells were sampled. (**b**) Examples of sections immunostained for BrdU and Hoechst from the rCX of an animal from each experimental group. These animals received one injection of BrdU on P2 and were sacrificed after 2 h. Calibration bars: (**a**) = 500 μm; (**b**) = 20 μm. (**c**–**h**) Total number of BrdU-positive cells in the rCX (**c**) and cCX (**d**), volume of the rCX (**e**) and cCX (**f**), and density of BrdU-positive cells in the rCX (**g**) and cCX (**h**) of untreated euploid (n = 8) and Ts65Dn (n = 7) mice and euploid (n = 7) and Ts65Dn (n = 9) mice treated with 7,8-DHF and in the cCX of untreated euploid (n = 9) and Ts65Dn (n = 6; n = 5 for the volume and density) mice and euploid (n = 7) and Ts65Dn (n = 11) mice treated with 7,8-DHF. Values (mean ± SE) in (**c**–**h**) refer to one hemisphere. The scatterplots over each column represent the values of individual cases for each group. *p < 0.05; **p < 0.01 (planned comparisons after one-way ANOVA). Images were acquired using NIS-Elements AR 4.30.02 (https://www.microscope.healthcare.nikon.com/it_EU/products/software/nis-elements/nis-elements-advanced-research). *7,8-DHF* 7,8-dihydroxyflavone, *CA1, CA3* hippocampal fields, *cCX* caudal cortex, *d* dorsal, *Eu* euploid, *l* lateral, *LV* lateral ventricle, *m* medial, *rCX* rostral cortex, *v* ventral, *Veh* vehicle.

**Figure 5:**
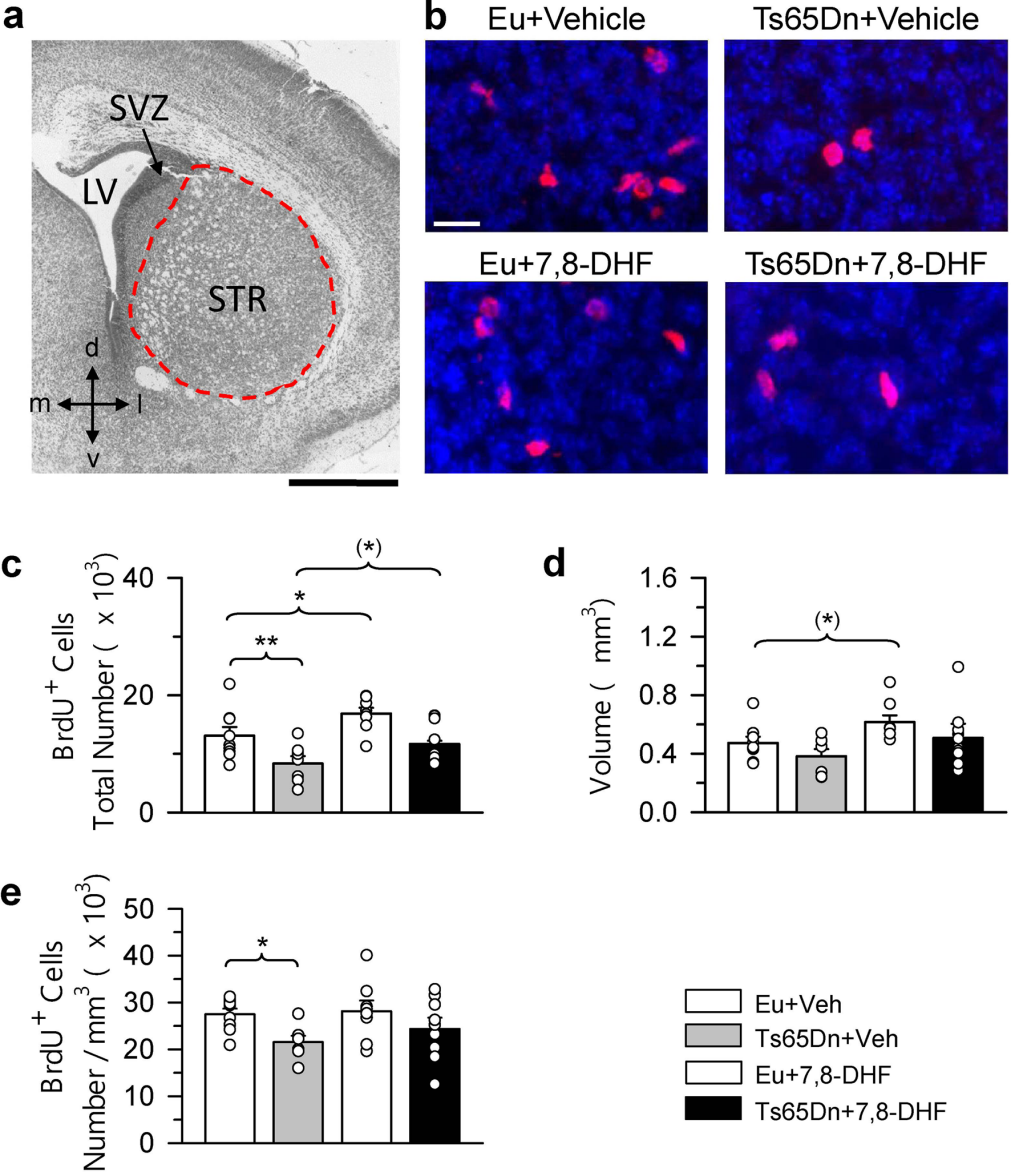
Effect of embryonic treatment with 7,8-DHF on proliferating cells in the striatum of P2 Ts65Dn and euploid mice. (**a**) Nissl-stained section across the striatum showing the region (area enclosed by a stippled line) where BrdU-positive cells were sampled. (**b**) Examples of sections immunostained for BrdU and Hoechst from the STR of an animal from each experimental group. These animals received one injection of BrdU on P2 and were sacrificed after 2 h. Calibration bars: (**a**) = 500 μm; (**b**) = 20 μm. (**c**–**e**) Total number of BrdU-positive cells in the STR (**c**), volume of the STR (**d**), and density of BrdU-positive cells in the STR (**e**) of untreated euploid (n = 9) and Ts65Dn (n = 7; n = 6 for the volume and density) mice and euploid (n = 8) and Ts65Dn (n = 10) mice treated with 7,8-DHF. Values (mean ± SE) in (**c**–**e**) refer to one hemisphere. The scatterplots over each column represent the values of individual cases for each group. *p < 0.06; *p < 0.05; **p < 0.01 (planned comparisons after one-way ANOVA). Images were acquired using NIS-Elements AR 4.30.02 (https://www.microscope.healthcare.nikon.com/it_EU/products/software/nis-elements/nis-elements-advanced-research). *Abbreviations 7,8-DHF* 7,8-dihydroxyflavone, *d* dorsal, *Eu* euploid, *l* lateral, *LV* lateral ventricle, *m* medial, *SVZ* subventricular zone, *v* ventral, *Veh* vehicle.

**Figure 6:**
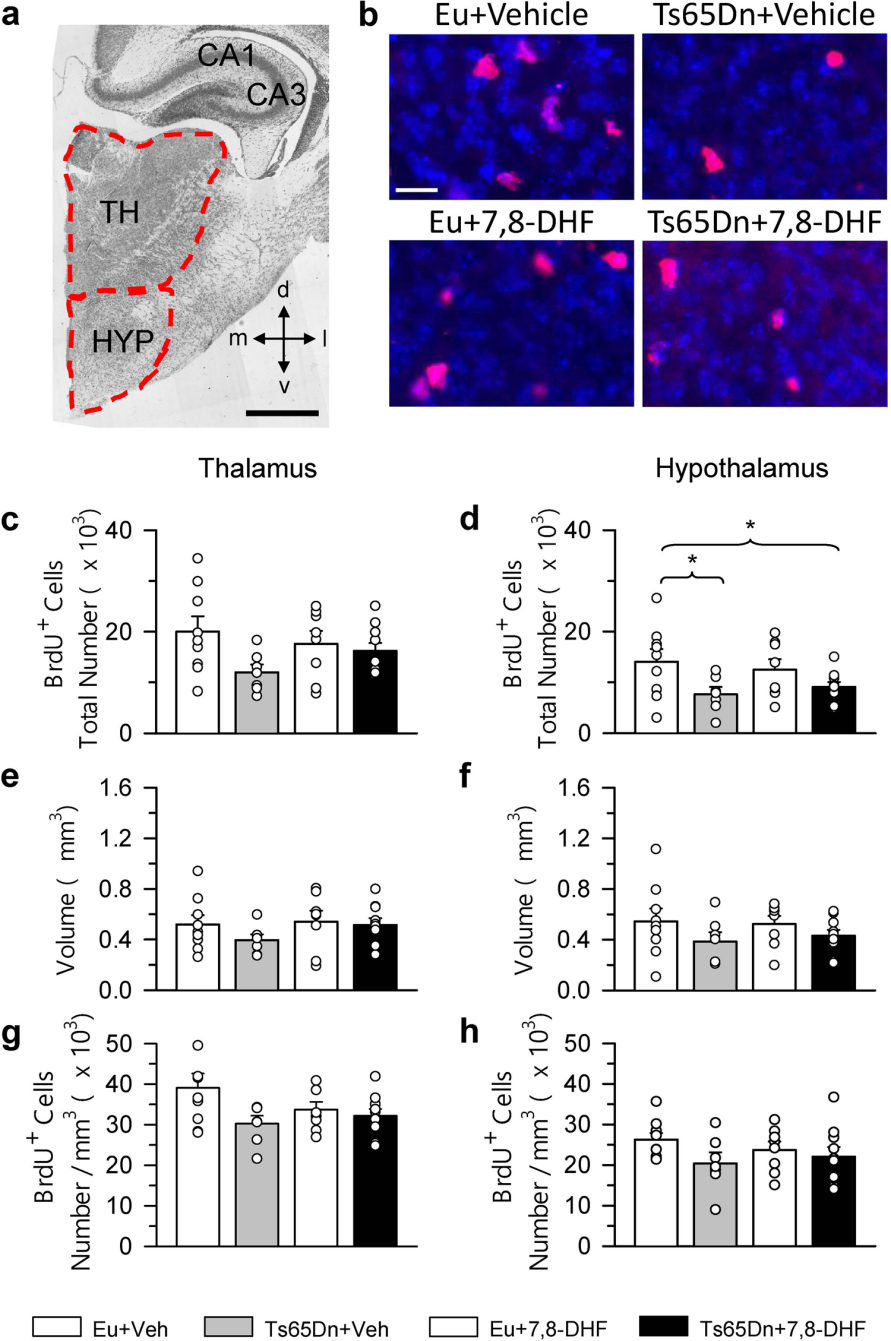
Effect of embryonic treatment with 7,8-DHF on proliferating cells in the thalamus and hypothalamus of P2 Ts65Dn and euploid mice. (**a**) Nissl-stained section across the thalamus and hypothalamus showing the region (areas enclosed by a stippled line) where BrdU-positive cells were sampled. (**b**) Examples of sections immunostained for BrdU and Hoechst from the TH of an animal from each experimental group. These animals received one injection of BrdU on P2 and were sacrificed after 2 h. Calibration bars: (**a**) = 500 μm; (**b**) = 20 μm. (**c**–**h**) Total number of BrdU-positive cells in the TH (**c**) and HYP (**d**), volume of the TH (**e**) and HYP (**f**), and density of BrdU-positive cells in the TH (**g**) and HYP (**h**) of untreated euploid (n = 9) and Ts65Dn (n = 7) mice and euploid (n = 8) and Ts65Dn (n = 10) mice treated with 7,8-DHF. Values (mean ± SE) in (**c**–**h**) refer to one hemisphere. The scatterplots over each column represent the values of individual cases for each group. *p < 0.05 (planned comparisons after one-way ANOVA). Images were acquired using NIS-Elements AR 4.30.02 (https://www.microscope.healthcare.nikon.com/it_EU/products/software/nis-elements/nis-elements-advanced-research). *Abbreviations 7,8-DHF* 7,8-dihydroxyflavone, *CA1, CA3* hippocampal fields, *d* dorsal, *Eu* euploid, * HYP* hypothalamus *l* lateral, *m* medial, *TH* thalamus *v* ventrl, *Veh* vehicle.

Neurons forming the brain derive from neural precursors surrounding the cerebral ventricles. These precursors are located in the ventricular zone (VZ) during early embryonic stages, and then in the subventricular zone (SVZ)^35^. Unlike the perinatal SVZ, that produces neurons and glia destined to the brain, the adult SVZ produces neurons specifically destined to the olfactory bulb. In the mouse, which is born 20–21 days after conception, most (90%) of the cell population of the SVZ is dividing at embryonic day 16 (E16), whereas the majority of the cells in the VZ are leaving the cell cycle. Neurons forming the neocortex are mainly born during the E11-E17 embryonic period and those forming the hippocampus are born between E10 and E18^36–38^. Most of the neurons forming the striatum, thalamus, and hypothalamus are born in the E11-E19 period^39–41^. Neurogenesis in the hippocampal dentate gyrus (DG) starts at E10, although in mice most of the granule neurons (about 80%) are generated by the postnatal subgranular zone (SGZ)^42^. In view of the time course of neurogenesis, we treated Ts65Dn mice with 7,8-DHF from E10 to birth, with the aim to establish whether treatment had a generalized restorative effect on neural precursor proliferation. This timing corresponds to that used in a previous study^22^, which will allow us to compare the effects of two different treatments on neurogenesis. In mice aged 2 days (called here P2 mice) we found numerous neural precursor cells in the SVZ and SGZ (Figs. 1, 2, 3; refer to Methods for definition of the SGZ of the current sudy). In addition, proliferating cells were scattered throughout the neocortex, striatum, thalamus, and hypothalamus (Figs. 4, 5, 6). The production of neurons and glia occurs at discrete time points during fetal brain development. Gliogenesis follows neurogenesis and persists long after neurogenesis has ceased. In rats, neurogenesis peaks at E14, astrocytogenesis at P2, and oligodendrocytogenesis at P14^43^. While proliferating cells in the neocortex, striatum, thalamus, and hypothalamus of P2 mice may include neuroblasts, they are likely to mainly represent future astrocytes and oligodendrocytes (glioblasts)^35^. These cells, that are derived from precursors in the VZ/SVZ retain proliferation capacity during migration and after they have reached their final destination^35^. To establish the possible efficacy of embryonic treatment with 7,8-DHF on the proliferation potency of neural precursors and their progeny, we evaluated the total number of BrdU-positive cells in the rostral and caudal SVZ (rSVZ; cSVZ), SGZ, rostral and caudal neocortex (rCX; cCX), striatum (STR), thalamus (TH) and hypothalamus (HYP) in embryonically treated P2 mice. We additionally evaluated the volume of the examined structures and estimated the density of proliferating cells (cells/mm^3^) in each examined region by dividing the total number of BrdU-positive cells by the volume.

### Effect of embryonic treatment with 7,8-DHF on proliferation potency in the SVZ of P2 Ts65Dn and euploid mice

A one-way ANOVA on the number of BrdU-positive cells in the rSVZ showed group differences [F(3,27) = 7.584, *p* = 0.001]. Planned comparisons (see Methods for the planned comparisons of this study) showed that untreated Ts65Dn mice had a reduced number of BrdU-positive cells (− 34%) in comparison with untreated euploid mice (Fig. 1c). In Ts65Dn mice treated with 7,8-DHF the number of BrdU-positive cells underwent an increase (+ 25%) in comparison with their untreated counterparts (Fig. 1c). However, this effect was not a restoration because treated Ts65Dn mice still had fewer BrdU-positive cells in comparison with euploid mice (Fig. 1c). In euploid mice treatment had no effect on the number of BrdU-positive cells (Fig. 1c). A one-way ANOVA on the volume of the rSVZ showed group differences [F(3,27) = 6.005, *p* = 0.003]. Planned comparisons showed that in untreated Ts65Dn mice the volume of the rSVZ was reduced (− 30%) in comparison with that of their untreated counterparts and that treatment increased the volume of the rSVZ that became similar to that of untreated euploid mice (Fig. 1d). In euploid mice treatment had no effect on the volume of the rSVZ (Fig. 1d). A one-way ANOVA on the density of BrdU-positive cells showed no significant differences between groups (Fig. 1e). A one-way ANOVA on the number of BrdU-positive cells in the cSVZ showed group differences [F(3,30) = 9.690, *p* < 0.001]. Planned comparisons showed that untreated Ts65Dn mice had a reduced proliferation potency (− 32%) in comparison with untreated euploid mice (Fig. 2c). In treated Ts65Dn mice the number of BrdU-positive cells underwent an increase (+ 45%) in comparison with their untreated counterparts (Fig. 2c). A comparison of untreated euploid mice and Ts65Dn mice treated with 7,8-DHF showed no difference between groups (Fig. 2c), indicating that treatment had restored the number of proliferating cells in the cSVZ. In euploid mice treatment caused an increase in the number of BrdU-positive cells that became larger (+ 29%) in comparison with that of untreated euploid mice (Fig. 2c). A one-way ANOVA on the volume of the cSVZ showed group differences [F(3,30) = 7.904, *p* > 0.001]. Planned comparisons showed that the volume of the cSVZ was reduced in untreated Ts65Dn (− 27%) in comparison with untreated euploid mice (Fig. 2d) and that this value was restored by treatment (Fig. 2d). Similarly to Ts65Dn mice, in euploid mice treatment caused an increase in the volume of the cSVZ (Fig. 2d). A one-way ANOVA on the density of BrdU-positive cells showed no significant differences between groups (Fig. 2e).

### Effect of embryonic treatment with 7,8-DHF on proliferation potency in the dentate gyrus of P2 Ts65Dn and euploid mice

A one-way ANOVA on the number of BrdU-positive cells in the DG showed group differences [F(3,28) = 5.258, *p* = 0.005]. Planned comparisons showed that in untreated Ts65Dn mice there were fewer BrdU-positive cells (− 25%) in comparison with their untreated euploid counterparts (Fig. 3b). In treated Ts65Dn mice there was an increase in the total number of BrdU-positive cells (+ 21%) that acquired a value similar to that of untreated euploid mice (Fig. 3b). In euploid mice, treatment with 7,8-DHF did not modify the number of proliferating cells in the DG (Fig. 3b). A one-way ANOVA on the volume of the DG showed group differences [F(3,26) = 4.304, *p* = 0.014]. Planned comparisons showed that the volume of the DG was reduced in untreated Ts65Dn (− 18%) in comparison with untreated euploid mice (Fig. 3c) and that this value was restored by treatment (Fig. 3c). In euploid mice treatment did not affect the volume of the DG (Fig. 3c). A one-way ANOVA on the density of BrdU-positive cells showed no significant differences between groups (Fig. 3d).

### Effect of embryonic treatment with 7,8-DHF on proliferation potency in the neocortex of P2 Ts65Dn and euploid mice

The Kruskal–Wallis test on the number of BrdU-positive cells in the rCX revealed a significant difference between groups [χ^2^(3) = 10.732, *p* = 0.013]. The Mann–Whitney test showed that untreated Ts65Dn mice had fewer proliferating cells (− 39%) in comparison with untreated euploid mice (Fig. 4c). The number of proliferating cells in treated Ts65Dn mice underwent an increase (+ 45%) in comparison with their untreated counterparts and became similar to that of untreated euploid mice (Fig. 4c). In euploid mice treatment had no effect on the number of BrdU-positive cells in the rCX (Fig. 4c). The Kruskal–Wallis test on the volume of the rCX revealed no significant difference between groups (Fig. 4e). A one-way ANOVA on the density of BrdU-positive cells in the rCX showed no differences between groups (Fig. 4g).

A one-way ANOVA on the number of BrdU-positive cells in the cCX showed group differences [F(3,29) = 7.188, *p* = 0.001]. Planned comparisons showed that untreated Ts65Dn mice had fewer proliferating cells (− 45%) in comparison with untreated euploid mice, but that treatment did not cause an increase in the number of proliferating cells in Ts65Dn mice in comparison with their untreated counterparts (Fig. 4d). A one-way ANOVA on the volume of the cCX showed group differences [F(3,28) = 3.039, *p* = 0.045]. Planned comparisons showed that untreated Ts65Dn mice had a reduced volume (− 19%) in comparison with untreated euploid mice but that volume was not affected by treatment (Fig. 4f). A one-way ANOVA on the density of BrdU-positive cells in the cCX showed group differences [F(3,28) = 9.073, *p* > 0.001]. Planned comparisons showed that untreated Ts65Dn mice had a reduced cell density (− 33%) in comparison with untreated euploid mice but that it was not affected by treatment (Fig. 4h).

### Effect of embryonic treatment with 7,8-DHF on proliferation potency in the striatum of P2 Ts65Dn and euploid mice

A one-way ANOVA on the number of BrdU-positive cells in the STR showed group differences [F(3,30) = 7.588, *p* = 0.001]. Planned comparisons showed that untreated Ts65Dn mice had fewer proliferating cells (− 36%) in comparison with untreated euploid mice (Fig. 5c). The number of proliferating cells in treated Ts65Dn mice underwent an increase (+ 40%) in comparison with their untreated counterparts and, although this effect was only marginally significant, the number of BrdU-positive cells became similar to that of untreated euploid mice (Fig. 5c). In euploid mice treatment caused an increase (+ 28%) in the number of proliferating cells in comparison with their untreated counterparts (Fig. 5c). A one-way ANOVA on the volume of the STR showed group differences [F(3,30) = 3.115, *p* = 0.041]. Planned comparisons showed that in treated euploid mice the volume of the STR became marginally larger than that of their untreated counterparts, but no differences in the other comparisons were observed (Fig. 5d). A one-way ANOVA on the density of BrdU-positive cells in the STR showed group differences [F(3,29) = 3.182, *p* = 0.039]. Planned comparisons showed that untreated Ts65Dn mice had a reduced density (− 22%) in comparison with untreated euploid mice; however, cell density in treated Ts65Dn mice was no higher than that of their untreated counterparts, although it did result to be statistically similar to that of untreated euploid mice (Fig. 5e).

### Effect of embryonic treatment with 7,8 DHF on proliferation potency in the thalamus and hypothalamus of P2 Ts65Dn and euploid mice

A one-way ANOVA on the number of BrdU-positive cells in the TH showed no group differences. However, Fig. 6c shows that in absolute terms the Ts65Dn mice had fewer cells in comparison with untreated euploid mice, which is in agreement with previous evidence^22^. A one-way ANOVA on the volume of the TH and density of BrdU-positive cells in the TH showed no group differences (Fig. 6e,g).

A one-way ANOVA on the number of BrdU-positive cells in the hypotalamus (HYP) showed marginal group differences [F(3,30) = 2.852, *p* = 0.054]. Planned comparisons showed that untreated Ts65Dn mice had a reduced number of proliferating cells in comparison with untreated euploid mice (− 45%; Fig. 6d) and that treatment did not increase the number of proliferating cells that remained lower (− 35%) in comparison with untreated euploid mice (Fig. 6d). A one-way ANOVA on the volume of the HYP and density of BrdU-positive cells in the HYP showed no group differences (Fig. 6f,h).

### Effect of embryonic treatment with 7,8-DHF on cellularity in P2 Ts65Dn and euploid mice

In view of the positive effect of treatment on proliferation potency in the SVZ and SGZ we wondered whether this effect translated into an increase in cell number. To this purpose, we evaluated the number of cells in the cCX, granule cell layer of the DG, and pyramidal layer of hippocampal field CA1.

#### Neocortex

**Figure 7:**
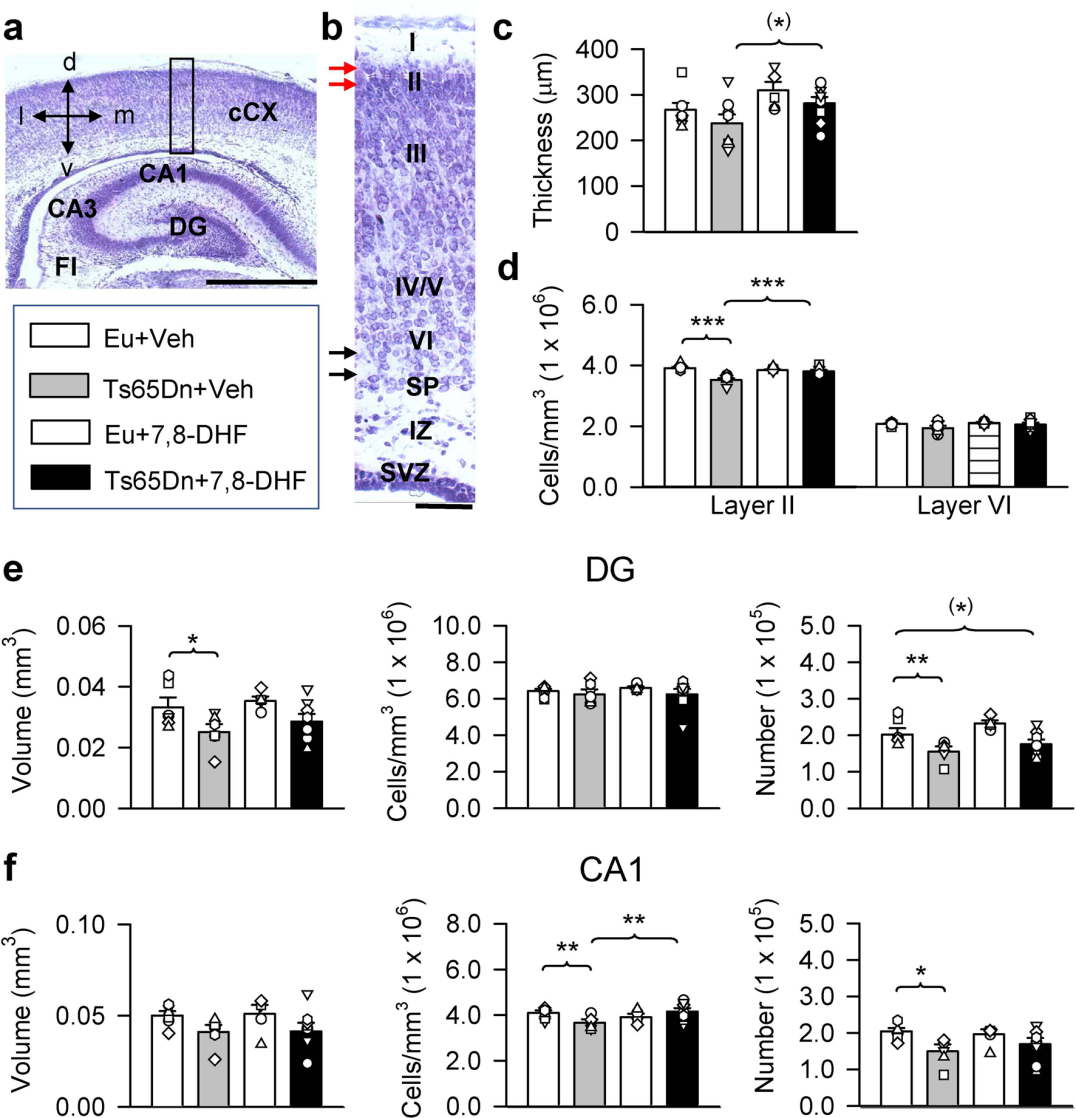
Effect of embryonic treatment with 7,8-DHF on the stereology of the neocortex and hippocampal region in P2 Ts65Dn and euploid mice. (**a**) Nissl-stained coronal section encroaching the cCX and the hippocampal formation. (**b**) Higher magnification of the region enclosed in a rectangle in (**a**) showing the cortical layers (ordinal numbers). The arrows indicate the regions where cells were counted in layer II (red arrows) and layer VI (black arrows). (**c**, **d**) Cortical thickness (layers II–VI) (**c**) and cell number (cells per mm^3^) in layer II and layer VI (**d**) in untreated euploid (n = 7) and Ts65Dn (n = 6 for layer II; n = 7 for layer VI) mice and euploid (n = 5) and Ts65Dn (n = 6 for layer II; n = 8 for layer VI) mice treated with 7,8-DHF. Calibration in (**a**) = 500 μm. Calibrations in (**c**) = 50 μm. (**e**, **f**) Volume of the granule cell layer, density of granule cells (number per mm^3^) and total number of granule cells of the DG (**e**), volume of the pyramidal layer, density of pyramidal neurons (number per mm^3^) and total number of pyramidal neurons of field CA1 (**f**) in untreated euploid (n = 6) and Ts65Dn (n = 6) mice and euploid (n = 5) and Ts65Dn (n = 8) mice treated with 7,8-DHF. Values (mean ± SE) in (**c**–**f**) refer to one hemisphere. The scatterplots over each column represent the values of individual cases for each group. *p < 0.06; *p < 0.05; **p < 0.01; ***p < 0.001 (planned comparisons after one-way ANOVA). Images were acquired using Image-Pro Plus version 4.5.0.29 (https://www.mediacy.com/imageproplus). *Abbreviations 7,8-DHF* 7,8-dihydroxyflavone, *CA1*, *CA3* hippocampal fields, *cCX* caudal cortex, *d* dorsal, *DG* dentate gyrus, *Eu* euploid, *FI* fimbria, *IZ* intermediate zone, *l* lateral, *m* medial, *SP* subplate, *SVZ* subventricular zone, *v* ventral, *Veh* vehicle.

A one-way ANOVA on the thickness of the cCX showed marginal group differences [F(3,24) = 2.899, *p* = 0.056]. Planned comparisons showed no difference between untreated Ts65Dn mice and euploid mice, although in absolute terms the cortical thickness of untreated euploid mice was larger in comparison with that of Ts65Dn mice (Fig. 7c). Treatment caused a marginal increase in the cortical thickness of Ts65Dn mice compared to their untreated counterparts but had no effect on cortical thickness in euploid mice (Fig. 7c). The development of the neocortex is characterized by an inside-outside pattern. Hence, the deep layers of the cortex are occupied by older neurons and the superficial layers are occupied by younger neurons. We deemed it of interest to establish the effect of treatment on cellularity in layer VI and layer II, because these layers contain neurons born at different times, the progenitors of which have been exposed to treatment for a shorter (progenitors of the deep neurons) and a longer (progenitors of the superficial neurons) period. A one-way ANOVA on the cell density in layer II showed group differences [F(3,20) = 14.310, *p* < 0.001]. Planned comparisons showed that in untreated Ts65Dn mice cell density was reduced (− 10%) in comparison with untreated euploid mice and that in treated Ts65Dn mice there was an increase in cell density (+ 8%) that acquired a value similar to that of untreated euploid mice (Fig. 7d). In treated euploid mice there was no change in cell density in layer II. Although data regarding the cell density of layer VI could not be statistically compared because the Kruskal–Wallis test did not show a significant effect, the graph in Fig. 7d clearly shows no differences between groups.

#### Dentate gyrus

A one-way ANOVA on the volume of the DG showed group differences [F(3,21) = 3.227, *p* = 0.043]. Planned comparisons showed that in untreated Ts65Dn mice the granule cell layer had a smaller volume in comparison with untreated euploid mice (Fig. 7e). The volume of the granule cell layer in Ts65Dn mice prenatally treated with 7,8-DHF became statistically similar to that of untreated euploid mice, although in absolute terms its size was smaller in comparison with that of euploid mice (Fig. 7e). The Kruskal–Wallis test showed no significant effect on granule cell density. A one-way ANOVA on total number of granule cells showed group differences [F(3,20) = 6.720, *p* = 0.003]. Planned comparisons showed that in untreated Ts65Dn mice the number of granule cells had a lower value in comparison with untreated euploid mice. Treatment with 7,8-DHF did not cause restoration of total granule cell number, which retained a lower value in comparison with that of untreated euploid mice (Fig. 7e).

#### Field CA1

A one-way ANOVA on the volume of the pyramidal layer of field CA1 showed no group differences [F(3,20) = 1.780, *p* = 0.183] (Fig. 7f), whereas a one-way ANOVA on the density of pyramidal neurons did show group differences [F(3,20) = 4.261, *p* = 0.018]. Planned comparisons showed that in untreated Ts65Dn mice cell density had a smaller value in comparison with untreated euploid mice (Fig. 7f). In Ts65Dn mice treated with 7,8-DHF there was an increase in cell density that became similar to that of untreated euploid mice (Fig. 7f). A one-way ANOVA on the number of pyramidal neurons showed marginal group differences [F(3,20) = 2.839, *p* = 0.060]. Planned comparisons showed that the number of pyramidal neurons of untreated Ts65Dn mice was reduced in comparison with that of untreated euploid mice (Fig. 7f). Although treatment did not significantly increase the total number of pyramidal neurons in Ts65Dn mice compared to their untreated counterparts, in absolute terms the number of pyramidal neurons in treated Ts65Dn mice became larger (169,341 ± 16,535) in comparison with untreated Ts65Dn mice (149,690 ± 19,064) and was statistically similar to that of untreated euploid mice (Fig. 7f).

### Effect of embryonic treatment with 7,8 DHF on BDNF expression in P2 Ts65Dn and euploid mice

Activation of the TrkB signaling pathway by 7,8-DHF has been shown to increase levels of BDNF in the brain^44–46^. BDNF is known to favor proliferation, differentiation, and migration of neuronal precursors cells^47–49^, suggesting that a treatment-induced increase in brain levels of BDNF may contribute to the positive impact of treatment on proliferation potency and cortical cell density in Ts65Dn mice. To clarify this issue, in P2 mice we evaluated the expression levels of BDNF in the cSVZ, an area that contains neural precursor cells destined to the cortex, and layer II of the cCX, that contains recently generated cortical neurons. A one-way ANOVA on the levels of BDNFin the cSVZ showed no group differences [F(3,8) = 0.768, *p* = 0.543] (Suppl. Figure 1b). Likewise, no group differences in the expression of BDNF were detected in layer II of the cCX [F(3,8) = 0.100, *p* = 0.958] (Suppl. Figure 1c).

### Long-term effect of embryonic treatment with 7,8-DHF on cell survival and neuronal phenotype acquisition in the dentate gyrus of adult Ts65Dn and euploid mice

**Figure 8:**
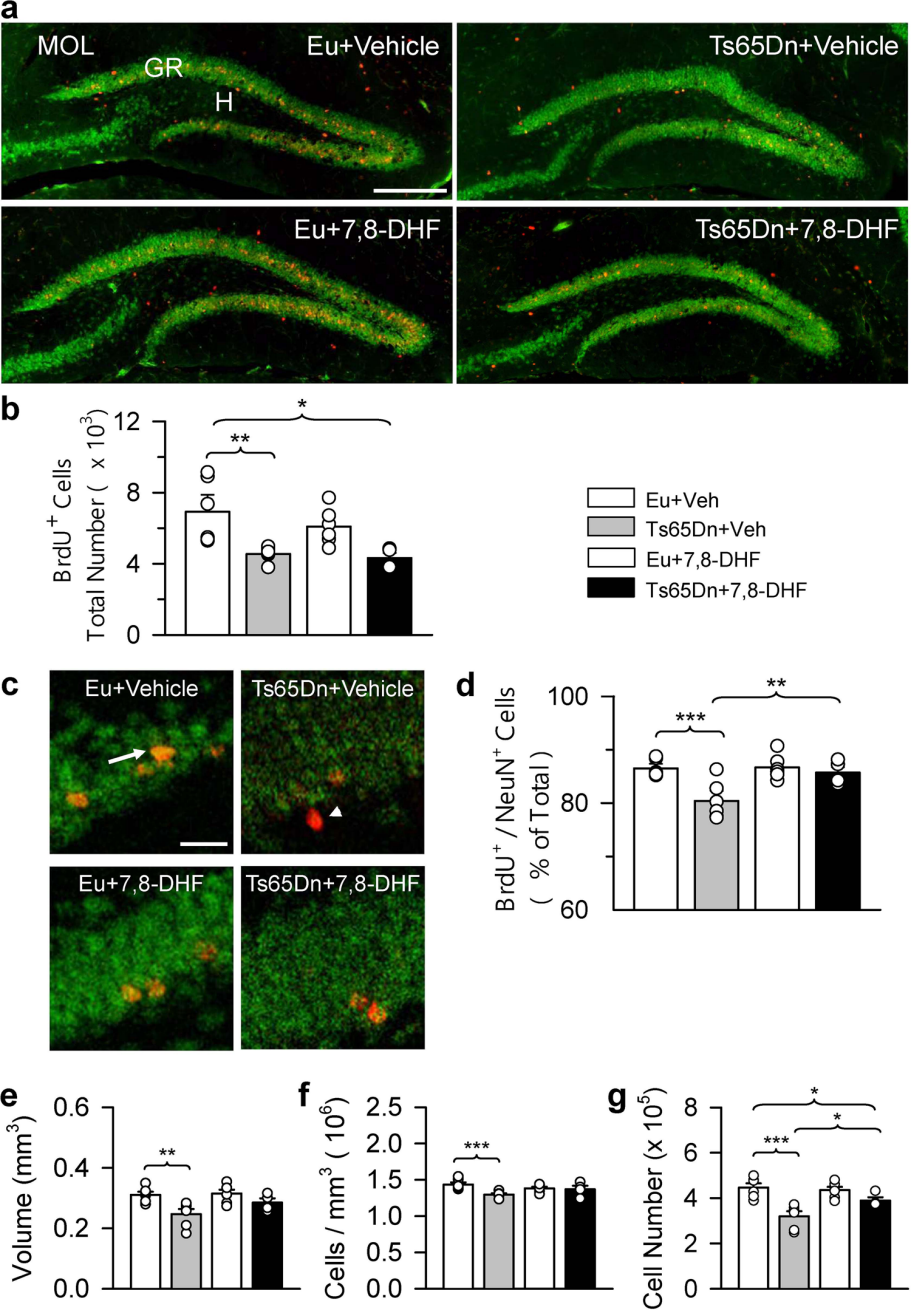
Effect of embryonic treatment with 7,8-DHF on neural precursor survival and neuronal phenotype acquisition in the dentate gyrus of adult Ts65Dn and euploid mice. (**a**) Examples of sections immunostained for BrdU and NeuN from the DG of an animal from each experimental group. These animals received one injection of BrdU on P2 and were sacrificed on P52–60 (adult mice). Calibration bar = 200 μm. Images were acquired using NIS-Elements AR 4.30.02 (https://www.microscope.healthcare.nikon.com/it_EU/products/software/nis-elements/nis-elements-advanced-research). (**b**) Total number of BrdU-positive cells in the hilus + subgranular zone + granule cell layer of the DG of untreated euploid (n = 6) and Ts65Dn (n = 6) mice and euploid (n = 6) and Ts65Dn (n = 4) mice treated with 7,8-DHF. (**c**) Examples of confocal images in the DG of an animal from each experimental group showing cells double-labeled for BrdU and NeuN (arrow), or BrdU only (arrow head). Calibration bar = 20 μm. (**d**) Percentage of cells double-labeled for BrdU and NeuN over total number of BrdU-positive cells. (**e**–**g**) Volume of the granule cell layer (**e**), density of the granule cells (**f**), and total number of granule cells (**g**) in the DG of untreated euploid (n = 6) and Ts65Dn (n = 6) mice and euploid (n = 6) and Ts65Dn (n = 4) mice treated with 7,8-DHF. Values in (**b**), (**d**), and (**e**–**g**) (mean ± SE) refer to one hemisphere. The scatterplots over each column represent the values of individual cases for each group. *p < 0.05; **p < 0.01; ***p < 0,001 (planned comparisons after one-way ANOVA). Images were acquired using Leica TCS, Leica Microsystems. (https://www.leica-microsystems.com/). *Abbreviations 7,8-DHF* 7,8-dihydroxyflavone, *DG* dentate gyrus, *Eu* euploid, *GR* granule cell layer, *H* hilus, *MOL* molecular layer, *Veh* vehicle.

Although neural progenitor cells multiply through a number of cell cycles, the final size of their progeny is limited because many of the new cells are bound to die. Cells born in the perinatal SVZ migrate to various neocortical areas and to the olfactory bulb^35^. In contrast, the final destination of cells born in the SGZ is the overlying granule cell layer. This restricted destination makes it easy to identify and quantify the progeny of SGZ precursor cells labeled at a given time point with a cell-cycle marker. Thus, in order to establish the long-term effect of prenatal treatment with 7,8-DHF on cell survival, we evaluated the number of BrdU-positive cells in the DG of mice injected with BrdU on P2 and sacrificed in adulthood (P52-P60; called herafter adult mice). In adult mice most of the labeled cells were located in the granule cell layer and only a few cells were still in the hilus (Fig. 8a). The Kruskal–Wallis test on the number of BrdU-positive cells revealed a significant difference between groups [χ^2^(3) = 15.251, *p* = 0.002]. The Mann–Whitney test showed that in untreated Ts65Dn mice the number of BrdU-positive cells was smaller in comparison with that of untreated euploid mice (Fig. 8b). Unlike at P2, in adult Ts65Dn mice prenatally treated with 7,8-DHF the number of BrdU-positive cells remained similar to that of their untreated counterparts (Fig. 8b). These findings indicate that although prenatal treatment with 7,8-DHF enhances the proliferation rate of the granule cell precursors of Ts65Dn mice, this effect does not translate into a long-term effect on their progeny. In order to establish whether prenatal treatment with 7,8-DHF affected the destiny of cells that had been born on P2 and survived into adulthood, we evaluated the percentage of cells that were co-labeled with BrdU and NeuN, a marker of mature neurons. A one-way ANOVA on the number of cells co-labeled with BrdU and NeuN showed group differences [F(3,18) = 8.215, *p* = 0.001]. Consistently with previous findings^11^, planned comparisons showed that in untreated Ts65Dn mice the percentage of cells that had differentiated into neurons was lower than that of untreated euploid mice (Fig. 8d). In treated Ts65Dn mice, however, there was an increase in the percentage of new neurons in comparison with their untreated counterparts and there were no differences in comparison with untreated euploid mice (Fig. 8d).

### Long-term effect of embryonic treatment with 7,8-DHF on granule neuron number in the dentate gyrus of adult Ts65Dn and euploid mice

We stereologically examined the DG in order to establish the long-term effect of embryonic treatment with 7,8-DHF on its size and total neuron number. A one-way ANOVA showed group differences in the volume of the DG [F(3,18) = 5.350, *p* = 0.008], granule cell density [F(3,18) = 4.760, *p* = 0.013], and total number of granule neurons [F(3,18) = 10.825, *p* > 0.001]. Planned comparisons showed that in untreated Ts65Dn mice the granule cell layer had a smaller volume (− 21%) than that of untreated euploid mice (Fig. 8e). The volume of the granule cell layer in treated Ts65Dn mice became statistically similar to that of untreated euploid mice, although in absolute terms its size was smaller in comparison with that of euploid mice (Fig. 8e). Granule cell density was reduced in untreated Ts65Dn mice in comparison with untreated euploid mice (− 10%); in treated Ts65Dn mice it became similar to that of euploid mice (Fig. 8f). In untreated Ts65Dn mice the number of granule cells was reduced (− 28%) in comparison with untreated euploid mice (Fig. 8g). In treated Ts65Dn mice there was an increase in the number of granule cells compared to their untreated counterparts, although the number remained lower than that found in untreated euploid mice (Fig. 8g).

### Effect of embryonic treatment with 7,8-DHF on pup viability and number

Ts65Dn females treated with 7,8-DHF during pregnancy did not exhibit a higher abortion rate in comparison with untreated females. The number of pups per litter and their survival rate was similar to that of their untreated counterparts (Table 1). This suggests that 7,8-DHF has no adverse effects on pregnancy and pup viability.

**Table 1:** Effect of embryonic treatment with 7,8-DHF on perinatal death and litter size.

Treatment	n	Spontaneous abortion		Litter size		Postnatal death	
		Mean	SE	Mean	SE	Mean	SE
Vehicle	10	0.00	0.00	5.00	0.44	4.04	2.78
7,8-DHF	10	0.14	0.14	5.61	0.47	5.72	3.26
*p*		n.s		n.s		n.s	

Pregnancy outcome for Ts65Dn females that were injected with either vehicle (n = 10) or 7,8-DHF (n = 10) from gestational day 10 to delivery. The number of spontaneous abortions of Ts65Dn females that received either vehicle or 7,8-DHF is expressed as the mean of total pregnancies of each group. The litter size is expressed as mean of individual litters. Postnatal death is expressed as the mean of the percentage of deaths over number of births in each litter. Data are mean ± SE. n.s. not significant (two-tailed t-test, for litter size; Mann–Whitney U test for spontaneous abortion and postnatal death).

### Effect of embryonic treatment with 7,8-DHF on body and brain weight in Ts65Dn and euploid mice

To establish the overall effect of prenatal treatment with 7,8-DHF we evaluated the body and brain weight of P2 and adult mice. A one-way ANOVA on the body weight of P2 mice showed group differences [F(3,33) = 4.519, *p* = 0.009]. In line with the well-known reduction in body weight exhibited by Ts65Dn mice^22,50^, both untreated and treated P2 Ts65Dn mice had a reduced body weight in comparison with euploid mice, although the difference was significant for treated Ts65Dn mice vs. untreated euploid mice (Table 2). Since the one-way ANOVA on brain weight showed no significant group differences [F(3,33) = 1.447, *p* = 0.247] data were not further analyzed. Table 2, however, shows that in absolute terms the brain weight of P2 Ts65Dn mice was smaller in comparison with their untreated counterparts which is in agreement with data from the literature^14,22,51,52^. A one-way ANOVA on body weight showed group differences [F(3,18) = 5.071, *p* = 0.010]. Planned comparisons showed that untreated Ts65Dn mice had a smaller body weight in comparison with untreated euploid mice (Table 2). No differences were found between treated Ts65Dn mice and untreated euploid mice. A one-way ANOVA on brain weight showed marginal group differences [F(3,18) = 2.788, *p* = 0.07]. Planned comparisons showed that the brain weight of adult Ts65Dn mice was smaller in comparison with their untreated counterparts which is in agreement with previous evidence^14^. Observation of Table 2 shows that treatment did not further reduce the body and brain weight of P2 or adult Ts65Dn mice, suggesting that treatment with 7,8-DHF has no adverse effects on either body growth or overall brain development.

**Table 2:** Effect of embryonic treatment with 7,8-DHF on body and brain weight.

	n	Mean ± SE		n	Mean ± SE	*p*
**P2 mice**						
*Body*						
Eu + Veh	9	1.87 ± 0.09	Eu + 7,8-DHF	8	2.02 ± 0.07	n.s
Ts65Dn + Veh	9	1.68 ± 0.16	Ts65Dn + 7,8-DHF	11	1.53 ± 0.06	n.s
*p*		n.s			< 0.01	
*Brain*						
Eu + Veh	9	0.12 ± 0.01	Eu + 7,8-DHF	8	0.12 ± 0.01	n.s
Ts65Dn + Veh	9	0.11 ± 0.01	Ts65Dn + 7,8-DHF	11	0.10 ± 0.01	n.s
*p*		n.s			n.s	
**Adult mice**						
*Body*						
Eu + Veh	6	20.69 ± 0.67	Eu + 7,8-DHF	6	19.23 ± 1.71	n.s
Ts65Dn + Veh	6	12.00 ± 1.98	Ts65Dn + 7,8-DHF	4	15.23 ± 2.97	n.s
*p*		0.002			n.s	
*Brain*						
Eu + Veh	6	0.44 ± 0.01	Eu + 7,8-DHF	6	0.44 ± 0.02	n.s
Ts65Dn + Veh	6	0.38 ± 0.02	Ts65Dn + 7,8-DHF	4	0.40 ± 0.02	n.s
*p*		0.028			n.s	

Body and brain weight (mean ± SE) in grams, of euploid and Ts65Dn mice used in the current study, measured on postnatal day 2 (P2) and P52–60 (adult mice). These mice received either vehicle (Veh) or 7,8-DHF in the embryonic period E10–E20/21. The *p* value in the row below the two genotypes refers to the comparison between untreated euploid (Eu + Veh) and Ts65Dn (Ts65Dn + Veh) mice and treated euploid (Eu + 7,8-DHF) and Ts65Dn (Ts65Dn + 7,8-DHF) mice. The *p* value in the column on the right refers to the comparison between untreated and treated mice of the same genotype. n.s. not significant (planned comparisons after one-way ANOVA).

## Discussion

Consistently with previous evidence^22^, we found a reduced proliferation potency in all (save for the TH) the examined forebrain regions of P2 Ts65Dn mice. We additionally found that in Ts65Dn mice the volume of the rSVZ, cSVZ, and SGZ was reduced in comparison with euploid mice. No differences were detected in the density of proliferating cells, suggesting that the reduction in the size of these proliferating niches is not further worsened by a reduction in the number of cells per unitary volume. Ts65Dn mice additionally exhibited a reduced volume of the STR, cCX, and HYP, which is line with the reduced size of the trisomic brain^53^. Embryonic treatment with 7,8-DHF had a beneficial effect in many of the examined regions of Ts65Dn mice. Treament increased the number of proliferating cells in both the rSVZ and cSVZ and increased the volume of both regions. The magnitude of the effect was different according to the rostro-caudal axis of the SVZ. While in the cSVZ of treated Ts65Dn mice the number of proliferating cells and the volume became similar to those of untreated euploid mice, this did not occur in the rSVZ, where, in absolute terms, the number of proliferating cells in treated Ts65Dn mice remained lower than in untreated euploid mice. In the SGZ of Ts65Dn mice treatment caused a large increase in the number of proliferating cells and volume that became similar to those of untreated euploid mice. In the rCX and STR of treated Ts65Dn mice the number of proliferating cells became statistically similar to that of untreated euploid mice although, in absolute terms, their number did not reach the value of that found in euploid mice. No volume increase, however, took place in these regions. At variance with the other examined regions, treatment did not increase the number of proliferating cells in the cCX, TH, or HYP.

The embryonic VZ/SVZ of the lateral ventricle is the neurogenic niche that harbors the progenitors of the neurons and glia forming the telencephalon (cerebral cortex, hippocampus and basal ganglia). The finding that there was a larger number of actively dividing cells at P2 in the SVZ of embryonically treated Ts65Dn mice in comparison with their untreated counterparts, indicates that embryonic treatment with 7,8-DHF had exerted a pro-proliferative effect on their progenitors. Since neurogenesis (i.e., production of neurons) takes place prenatally, this suggests, in turn, a positive effect of embryonic treatment with 7,8-DHF on telencephalic neurogenesis. In the early postnatal period, the SVZ of the lateral ventricle gives origin to glial cells (initially astrocytes, and then oligodendrocytes) destined to the cortex and striatum^35^. The reduced number of proliferating cells in the CX and STR observed here in untreated Ts65Dn mice is consistent with the reduced proliferation potency in their SVZs, and the increase in the number of these cells in treated Ts65Dn mice is consistent with the treatment-induced increase in the number of progenitors in the SVZ. Unlike in the rCX, treatment did not increase the number of proliferating cells in the cCX. The number of cells that migrate away from the germinative zones of the brain depends not only on the proliferation potency of the progenitors but also on attractive/repulsive chemical signals arising from the cellular environment. The molecular processes regulating astrocyte and oligodendrocyte precursor migration in DS remain poorly defined. The finding that treatment did not increase the number of glioblasts in the cCX may be explained in terms of lack of signals favoring their migration.

The SGZ is the specific source of neurons (and astrocytes) forming the DG. The reduced number of proliferating cells in the SGZ of P2 Ts65Dn mice indicates that precursor proliferation is impaired starting from the earliest phases of DG development. Treatment fully restored the number of progenitors in the SGZ of Ts65Dn mice, indicating that the progenitors of the SGZ are sensitive to the effects of 7,8-DHF not only in the neonatal period^14^ but also during the embryonic period.

The progenitors of cells forming the diencephalon (thalamus and hypothalamus) are located in the VZ/SVZ lining the III ventricle^54^. The finding that at P2 untreated Ts65Dn mice had fewer proliferating cells in the TH and HYP suggests that this reduction may be due to impairment of precursor proliferation in the VZ/SVZ of the III ventricle. Two reports provide evidence for a reduction in the number of neurons and glial cells in thalamic nuclei of aged people with DS^55,56^. While neurodegeneration may contribute to this reduction it is also conceivable that early neurogenesis alterations may underlie the reduced number of thalamic neurons. We found that treatment did not increase the number of proliferating cells in the TH and HYP of Ts65Dn mice, suggesting that treatment does not exert beneficial effects on diencephalic progenitors.

Taken together, these results show that prenatal treatment with 7,8-DHF exerts widespread pro-proliferative effects in the forebrain, although these effects may vary in magnitude according to the brain region. 7,8-DHF specifically binds to the TrkB receptor, mimicking the action of the natural agonist, BDNF. Since activation of this pathway enhances BDNF production^44–46^ we focused here on BDNF as a potential mediator of the treatment-induced proliferation enhancement in Ts65Dn mice. In contrast to the differences found at older ages^57^, we found no differences in BDNF expression between Ts65Dn and euploid pups. Moreover, treatment did not significantly change BDNF levels, suggesting that other mechanisms may underlie proliferation enhancement in prenatally treated Ts65Dn pups. According to previous evidence, 7,8-DHF may influence proliferation in a non-cell autonomous manner^14^, suggesting that numerous mechanisms may underlie the pro-proliferative effects of 7,8-DHF. Accordingly, the environment of the neurogenic niche and/or of the final destination of migrating neuroblasts/glioblasts may explain the differences in the strength and distribution of the pro-proliferative effect of 7,8-DHF observed here.

Untreated Ts65Dn mice exhibited a reduced cellularity in layer II, but not in layer VI of the cCX, which is partially in agreement with our previous findings^22^. Evidence that the superficial cortical layers are more liable to being compromised in comparison with deep layers in fetuses and infants with DS^58–62^ is consistent with the current finding of a reduced cell density in layer II but not in layer VI of the Ts65Dn models of DS. Prenatal treatment with 7,8-DHF restored cell density in layer II of Ts65Dn mice, which is consistent with its pro-proliferative effect in the cSVZ. In contrast, treatment had no effect in layer VI of the cCX. Corticogenesis follows an inside-outside pattern and earlier born neurons settle into the deeper laminae while later born neurons migrate past them to colonize more superficial layers. The VZ forms the projection neurons in the deeper layers of the neocortex, while the SVZ forms the projection neurons in the outer layers^35^. In mice, corticogenesis begins approximately on E10, i.e., at the time when we initiated treatment. This implies that at the time at which layer II neurons were born their progenitors had been exposed to treatment for a longer period in comparison with the progenitors giving origin to layer VI neurons. The necessity of a relatively long exposure to treatment for the improvement of proliferation may explain the positive effect of treatment on the cellularity of layer II but not of layer VI.

Since the hippocampus is damaged in DS and plays a critical role in learning and memory, we were interested in establishing whether prenatal treatment with 7,8-DHF improves its development. Consistently with previous evidence, we found that Ts65Dn mice had a reduced number of neurons in the DG and field CA1^22^. Treatment did not increase total cell number in the DG of P2 Ts65Dn mice although it attenuated the difference in comparison with untreated euploid mice. Thus, although treatment restored the number of progenitors of the granule neurons in the SGZ, this effect was not sufficient to restore the number of granule neurons. Regarding the hippocampus, although treatment restored cell density in field CA1 of Ts65Dn mice, the total number of pyramidal neurons did not reach, in absolute terms, the value of untreated euploid mice. Taken together, these results show that prenatal treatment with 7,8-DHF improves cortical cellularity but has very moderate effects on the development of the hippocampal formation of Ts65Dn mice. Regarding the long-term effects of embryonic treatment with 7,8-DHF, our study shows no enhancing effect on the survival of cells born on P2 in the DG of adult Ts65Dn mice (P52–60 mice). In these mice, however, there was an increase in the percentage of BrdU-positive cells that had acquired a neuronal phenotype (BrdU/NeuN-positive cells) and in the number of granule neurons forming the DG, although their number remained lower than that of euploid mice. These findings indicate that the pro-proliferative effects of embryonic treatment with 7,8-DHF translate into a long-term, albeit moderate, effect on DG neurogenesis.

A comparison of the effects of prenatal treatment with 7,8-DHF observed here with the effects of prenatal treatment with fluoxetine observed in our previous study^22^ shows that fluoxetine exerts remarkably more prominent effects than 7,8-DHF. Fluoxetine fully restored the number of proliferating cells in the rSVZ, cSVZ, SGZ, rCX, cCX, STR, TH, and HYP, as well other brain regions not examined here. In addition, fluoxetine restored cellularity in all cortical layers, in the DG and the hippocampus. Moreover, while the effects of prenatal treatment (current study) and of neonatal treatment^63^ with 7,8-DHF leave a modest trace (current study) or disappear^63^ after treatment cessation, the effects of either pre- or postnatal treatment with fluoxetine are retained well after treatment discontinuation and translate into a behavioral restoration in adulthood^11,12,22^. Thus, fluoxetine is more advantageous for the trisomic brain in comparison with 7,8-DHF. Yet, as mentioned above, fluoxetine is an antidepressant that may pose caveats for human use during pregnancy due to unwanted side effects, whereas, flavonoids, in view of their chemical nature, are potentially usable during gestation. Thus, weighing up the pros and cons of treatment with either flavonoids or a more effective molecule such as fluoxetine for the treatment of DS, the former may be preferable despite their milder effects.

In conclusion, the current study in a DS model shows that embryonic treatment with 7,8-DHF restores/improves the proliferation potency of numerous brain neurogenic niches but has mild long-term effects. Previous evidence showed that the effects of treatment with 7,8-DHF in the neonatal period disappear if treatment is interrupted^14,63^. It remains to be established whether a treatment with 7,8-DHF that starts during pregnancy and continues in the early postnatal period can lead to an improvement in brain development that is retained in adulthood.

## Materials and methods

### Colony

Ts65Dn mice were generated by mating B6EiC3Sn a/A-Ts(17^16)65Dn females with C57BL/6JEiJ x C3H/HeSnJ (B6EiC3Sn) F1 hybrid males. This parental generation was provided by Jackson Laboratories (Bar Harbor, ME, USA). To maintain the original genetic background, the mice used were of the first generation of this breeding. Animals were genotyped as previously described^64^. The day of birth was designated postnatal day zero (P0). The animals’ health and comfort were controlled by the veterinary service. The animals had access to water and food ad libitum and lived in a room with a 12:12 h light/dark cycle. Experiments were performed in accordance with the European Communities Council Directive of 24 November 1986 (86/609/EEC) for the use of experimental animals and were approved by Italian Ministry of Public Health (Protocol n. 205/2019-PR). In this study, all efforts were made to minimize animal suffering and to keep the number of animals used to a minimum.

### Experimental protocol

Ts65Dn females (n = 20) were bred with C57BL/6JEi x C3SnHeSnJ (B6EiC3Sn) F1 males (n = 12). Conception was determined by examining the vaginal plug. Pregnant females received a daily subcutaneous injection of either 7,8-dihydroxyflavone (7,8-DHF, Sigma Aldrich) in vehicle (dose: 5 mg/kg) (n = 10) or vehicle (PBS with 2% DMSO) (n = 10) from embryonic (E) day 10 (E10) to the day of birth (E20/21). The progeny of females that received 7,8-DHF will hereafter be called “treated mice” whereas the progeny of females that received the vehicle will be called “untreated mice”. On postnatal day 2 (P2) the progeny of treated (10 litters) and untreated (10 litters) females received an intraperitoneal injection (150 μg/g body weight) of BrdU (Sigma Aldrich) in TrisHCl 50 mM and were killed either after 2 h (called here P2 mice) or after 52–60 days (called here adult mice). Since the Ts65Dn strain is characterized by a high mortality rate during gestation and before weaning, the number of Ts65Dn pups per litter is approximately one third instead than one half. Measurements were carried out in a total of 37 P2 mice and 22 adult mice deriving from all litters, approximately balanced in number per treatment and strain. Body weight was recorded prior to sacrifice. After sacrifice, the brain was excised and weighed.

### Histological procedures

Histological procedures were carried out as previously described^11,22,65^. P2 animals were decapitated and the brain was removed. The rostral brain (forebrain plus mesencephalon; called hereafter hemisphere) was separated from the hindbrain (cerebellum plus pons and medulla), cut along the midline and fixed by immersion in Glyo-Fixx. Each hemisphere was dehydrated through a series of ascending ethanol concentrations, embedded in paraffin, and cut in series of 8-µm-thick coronal sections. One out of every 20 sections through the entire hemisphere was attached to a poly-lysine coated slide and used for BrdU immunohistochemistry (right hemisphere) and Nissl-staining (left hemisphere). Adult mice were killed with an overdose of Isoflurane 2%, and their brains were removed. The rostral brain was cut along the midline, fixed by immersion in PFA 4%, and frozen. The entire right hemisphere was cut with a freezing microtome into 30-μm-thick coronal sections that were serially collected in anti-freezing solution (30% glycerol; 30% ethylen-glycol; 10% PBS10X; sodium azide 0.02%; MilliQ to volume). The number of mice used for the histological procedures was 8–11 per group in P2 mice and 4-6 per group in adult mice (Table 3).

**Table 3:** Number of embryonically treated mice used in the current study.

	Males	Females	Total
**P2**			
Eu + Veh	5	4	9
Ts65Dn + Veh	7	2	9
Eu + 7,8-DHF	4	4	8
Ts65Dn + 7,8-DHF	3	8	11
**P52–60**			
Eu + Veh	4	2	6
Ts65Dn + Veh	2	4	6
Eu + 7,8-DHF	3	3	6
Ts65Dn + 7,8-DHF	2	2	4

Number and sex of euploid and Ts65Dn mice treated with either vehicle or 7,8-DHF used in the current study. *7,8-DHF* 7,8-dihydroxyflavone, *Veh* vehicle.

#### BrdU immunohistochemistry

Sections from the whole right hemisphere of P2 mice and sections from the right hippocampal formation of adult mice were incubated with a primary rat anti-BrdU antibody (diluted 1:200; Biorad) and a mouse monoclonal anti-NeuN (Neuronal-specific nuclear protein), a marker of mature neurons (diluted 1:250, Chemicon, Billerica, MA, USA). Detection was performed using a Cy3-conjugated anti rat-secondary antibody for BrdU immunohistochemistry (diluted 1:200; Jackson Immunoresearch) and a FITC-conjugated anti-mouse IgG for NeuN immunohistochemistry (diluted 1:200; Jackson Immunoresearch). Sections were additionally incubated for 2 min in Hoechst nuclear dye (0.2 mg/ml in PBS). The penetration of the anti-BrdU antibody was checked through 3-D image reconstruction (see Supplementary Fig. 2).

#### BDNF immunohistochemistry

Sections taken at the level of the cCX of P2 mice were subjected to BDNF immunohistochemistry as detailed in Supplementary Methods.

#### Nissl-staining

Sections from the left hemisphere of P2 and adult mice were stained with Toluidine Blue according to the Nissl method.

### Measurements

#### Number of BrdU-positive cells

BrdU-positive cells were sampled in the subventricular zone (SVZ), dentate gyrus (DG), neocortex (CX), striatum (STR), thalamus (TH), and hypothalamus (HYP) of P2 mice and from the DG af adult mice. The brain coordinates (BC) reported below for these regions in P2 mice refer to the "Atlas of the developing mouse brain"^66^. Since different regions of the perinatal VZ/SVZ give origin to neurons destined to different telencephalic areas^35^, we evaluated the effects of treatment in two different rostro-caudal regions of the SVZ separately, named here as follows: the rostral SVZ (rSVZ) is the region that stretches from the rostral horn of the lateral ventricle to the beginning of the hippocampal formation (BC: 2.19–3.15 mm) and the caudal SVZ (cSVZ) is the region that stretches from the beginning to the end of the hippocampal formation (BC: 3.27–4.84 mm). While in the adult DG the proliferating niche is formed by cells that are located almost exclusively in the subgranular zone (SGZ), in P2 pups BrdU-positive cells were scattered throughout the hilus, SGZ, and granule cell layer (see Fig. 3a). For the sake of simplicity, here these regions are collectively called SGZ. In the DG of P2 pups, we counted all BrdU-positive cells along the whole rostro-caudal extent of this region (BC: 3.27–4.84 mm). In adult mice that had received BrdU on P2, most of the BrdU-positive cells had migrated to the granular layer and fewer cells were still in the SGZ and hilus (see Fig. 8a). In these mice we counted the BrdU-positive cells present in the granule cell layer plus SGZ plus hilus along the whole rostro-caudal extent of the DG. In the CX, BrdU-positive cells were separately evaluated in the CX overlying the rSVZ (rostral cortex, rCX; BC: 2.19–3.15 mm) and the cSVZ (caudal cortex, cCX; BC: 3.27–4.84 mm). Cells in the STR were counted in sections that encroached the rSVZ (BC: 2.19–3.15 mm). In the TH and HYP cells were counted in sections comprised between the BC 3.27–4.23 and the BC 2.67–3.87 mm, respectively. In each region of interest, BrdU-positive cells were counted within the areas indicated in Figs. 1a, 2a, 3a, 4a, 5a, and 6a. BrdU-positive cells were detected using a fluorescence microscope (Eclipse; objective: × 20, 0.5 NA). Quantification of BrdU-labeled nuclei was conducted in every 20th section in P2 mice and every 6th section in adult mice. Using this spacing ensures that the same neuron will not be counted in two sections. The number of sampled sections for each region of interest is reported in Suppl. Table 1. These sections cover the whole rostro-caudal extent of the region of interest. Cell counting was carried out using a modified stereology protocol^67–69^. In each section the borders of the region of interest were first manually traced and all BrdU-labeled cells located in this region were counted. The total number of BrdU-labeled cells counted in the series of sampled sections was multiplied by 20 in P2 mice and by 6 in adult mice (the inverse of the section sampling fraction: 1/20 and 1/6, respectively) to obtain the total estimated number of BrdU-positive cells per each region of interest. In P2 mice, we evaluated the volume of the region of interest by multiplying the sum of the cross-sectional areas traced in individual sections by the spacing T between sampled sections (160 μm). The estimated total number of BrdU-positive cells was divided by the volume of the region of interest in order to obtain the density (cells/mm^3^) of BrdU-labeled cells for each region.

#### Neuronal phenotype acquisition

In sections from the DG of adult mice taken with a confocal microscope (Leica TCS, Leica Microsystems, Wetzlar, Germany) we counted the number of cells that co-expressed BrdU and NeuN, a marker of mature neurons. The number of cells that exhibited a neuronal phenotype was expressed as a percentage of the total number of BrdU-positive cells.

#### Stereology

Stereology of the DG and field CA1 was conducted in Nissl-stained sections of P2 mice, starting from a random position of the first (rostralmost) section plane; in adult mice stereology was carried out in the DG. The volume of the granule cell layer of the DG, pyramidal layer of field CA1, cell numerical density (De), and total number of neurons in the DG and CA1 were estimated as previously described^11,70,71^ and summarized as follows. In each sampled section the areas of the granule cell layer of the DG and of the pyramidal layer of field CA1 were measured by tracing their contours. The volume (Vref) of the granule cell layer and pyramidal layer were estimated based on Cavalieri’s method^70,72^, that is, by multiplying the sum of the cross-sectional areas by the spacing T between sampled sections (160 μm  in P2 mice and 180 μm in adult mice). Cell numerical density was determined with the optical disector method using systematic disector samples. Counting frames (disectors) with a side length of 15 μm and a height of 8 μm, in P2 mice, and 30 μm, in adult mice, spaced in a 100 μm square grid (fractionator) were systematically used. Cell nuclei were counted with a × 100 oil objective (1.4 NA). Cell nuclei that intersected the uppermost focal plane or intersected the exclusion lines of the count frame were not counted.

Neuron density (De) is given by

$${\text{De}} = \left( {\sum {\text{Q}} /\sum {{\text{dis}}} } \right)/{\text{Vdis}}$$

where Q is the number of particles counted in the disectors, dis is the number of disectors and Vdis is the volume of the disector. Calculation of CE of De gave values between 0.051 and 0.069.

The total number (N) of cells in the granule cell layer of the DG and pyramidal layer of field CA1was estimated as the product of Vref and the numerical density (De).

$${\text{N}} = {\text{De}} \times {\text{Vref}}$$

In the cortex overlying field CA1 (cCX) we evaluated cortical thickness and cell cell density in layer II and VI (Fig. 7a). In layer II cells were counted in the portion of the layer close to layer I, and in layer VI cells were counted in the portion of the layer close to the subcortical plate (Fig. 7b). The thickness of the cCX overlying field CA1 was measured by tracing radial lines across the cellular layers II-VI at 4–5 locations. The De in layers II and VI was evaluated using counting frames with a side length of 15 μm (in layer II) and 20 μm (in layer VI) and a height of 8 μm spaced in a 100 μm square grid. Cell density was expressed as number of cells/mm^3^.

The number of mice used for each procedure is reported in the figure legends. Supplementary Table 2 summarizes the number of P2 mice used for evaluation of the number of BrdU-positive cells and stereology, respectively, in each region of interest. Note that the number of mice used for measurements in different regions may vary due to technical reasons (rupture of some sections or poor staining quality). All measurements were carried out by an experimenter blinded to the study code.

#### BDNF expression levels

BDNF expression levels in the cSVZ and cCX of P2 mice were evaluated as detailed in Supplementary methods.

### Statistical analysis

Results are presented as mean ± standard error of the mean (SE). Data were analyzed with the IBM SPSS 22.0 software. Before running statistical analyses, we checked data distribution and homogeneity of variances for each variable using the Shapiro–Wilk test and Levene’s test, respectively. If the data were normally distributed and variance was homogeneous, statistical analysis was carried out using a one-way ANOVA followed by a priori planned comparisons^73^. We compared untreated Ts65Dn mice to untreated euploid mice, treated Ts65Dn mice to untreated Ts65Dn mice, treated Ts65Dn mice to untreated euploid mice, and treated euploid mice to untreated euploid mice. If the data were not normally distributed and variance was heterogeneous, transformations were made to achieve normality. If the transformed data did not achieve normality, statistical analysis was carried out using the Kruskal–Wallis test followed by the Mann–Whitney U test. Based on the “Box plot” tool available in SPSS Descriptive Statistics, in each analysis we excluded the extremes, i.e., values that were larger than 3 times the IQ range [x ≥ Q3 + 3 * (IQ); x ≤ Q1 – 3 * (IQ)]. A probability level of *p* ≤ 0.05 was considered to be statistically significant.

### Data availability

The dataset is available from the corresponding authors upon reasonable request.

## Supplementary Information

**Supplementary Information 1.**

## Abbreviations

7,8-DHF: 7,8-dihydroxyflavone
BDNF: Brain-derived neurotrophic factor
CA1: Hippocampal field
CA3: Hippocampal field
CX: Cortex
cCX: Caudal cortex
cSVZ: Caudal subventricular zone
d: Dorsal
DG: Dentate gyrus
DS: Down syndrome
E: Embryonic
Eu: Euploid
FI: Fimbria
HYP: Hypothalamus
IZ: Intermediate zone
l: Lateral
LV: Lateral ventricle
m: Medial
p: Postnatal day
rCX: Rostral cortex
rSVZ: Rostral subventricular zone
SGZ: Subgranular zone
SP: Subplate
STR: Striatum
SVZ: Subventricular zone
TH: Thalamus
TrkB: Tropomyosin-related kinase B
v: Ventral
Veh: Vehicle
VZ: Ventricular zone
