# Supplementary material for: The flavonoid 7,8-DHF fosters prenatal brain proliferation potency in a mouse model of Down syndrome
Source: Sci Rep. 2021 Mar 18;11:6300. doi: 10.1038/s41598-021-85284-5 (PMC7973813; doi:10.1038/s41598-021-85284-5)
Supplement: Supplementary file 1 — Supplementary Information 1. [file 41598_2021_85284_MOESM1_ESM.pdf]

Title: The flavonoid 7,8-DHF fosters prenatal brain proliferation potency in a mouse model of Down syndrome

Authors: Fiorenza Stagni [a], Beatrice Uguagliati [b], Marco Emili [b], Andrea Giacomini [b], Renata Bartesaghi [b], Sandra Guidi [b]

Address:

a) Department for Life Quality Studies, University of Bologna, Rimini, Italy.

b) Department of Biomedical and Neuromotor Sciences, University of Bologna, Bologna, Italy.

SUPPLEMENTARY INFORMATION

SUPPLEMENTARY METHODS

BDNF immunohistochemistry. Sections (n = 4-6 per animal) encroaching the cSVZ and cCX overlying the hippocampus of P2 mice were submitted to immunohistochemistry for BDNF. Sections were incubated for 24 h at 4°C with mouse monoclonal anti-BDNF antibody (mouse monoclonal anti-BDNF, Clone 35928.11, Sigma-Aldrich; 1:200). Sections were then incubated with biotinylated anti-mouse secondary antibody (anti-mouse IgG biotin conjugate; Sigma-Aldrich) for 2 h. The antibody binding sites were visualized through the reaction with diaminobenzidine-H_2O_2 solution after incubation with avidin-biotin complex (Vector Laboratories, Inc., Burlingame, CA, USA; BA-4001).

Evaluation of BDNF expression levels. The intensity of BDNF immunoreactivity in the cSVZ and layer II of the cCX neocortex was determined by optical densitometry of immunohistochemically-stained sections. Images were captured using an optical microscope (objective x 25, final magnification x 250). Densitometric analysis was carried out using the software Image Pro Plus. A box of 155 µm^2 was randomly placed on the cSVZ and a box of 260 µm^2 was randomly placed on layer II of the cCX. Twenty measurements were taken for each region in each sampled section. For each image, the intensity threshold was estimated by analyzing the distribution of pixel.

SUPPLEMENTARY FIGURES

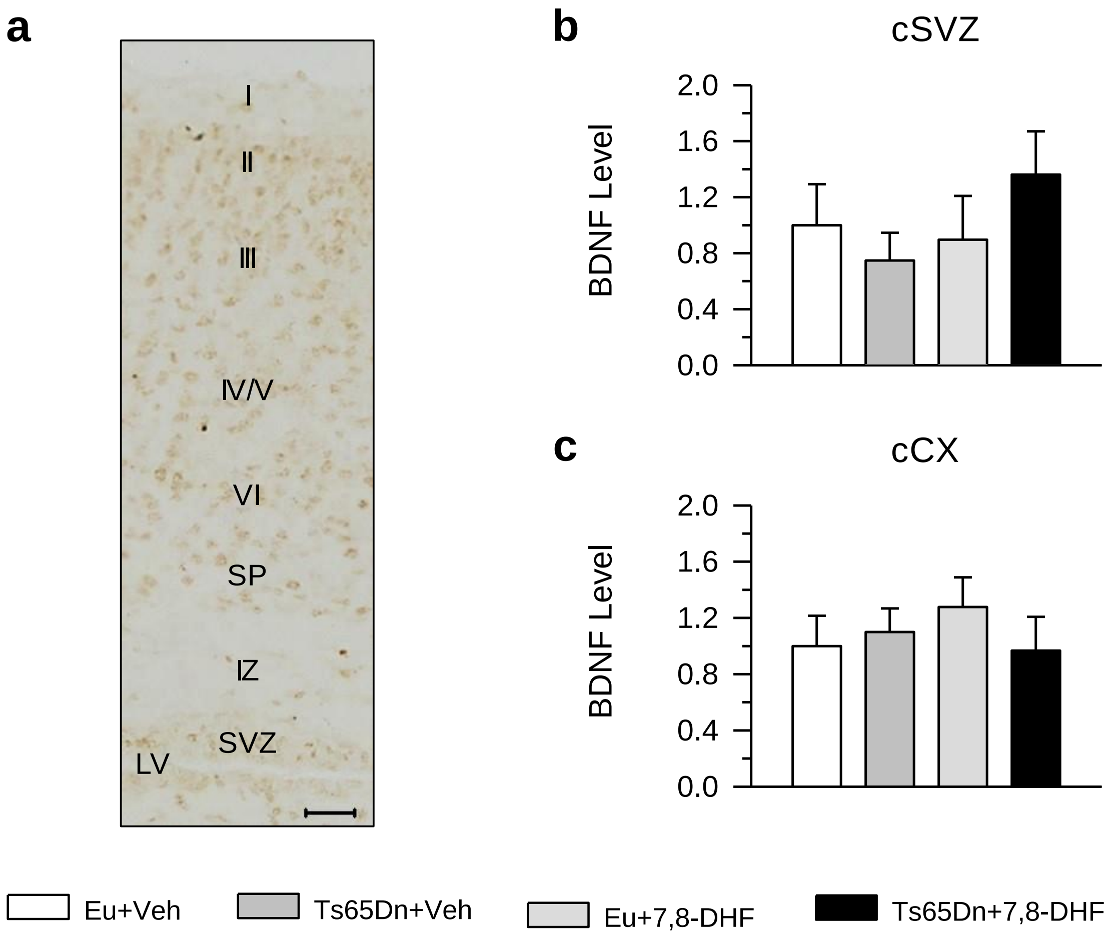

Suppl. Fig. 1. Effect of embryonic treatment with 7,8-DHF on BDNF expression levels in P2 Ts65Dn and euploid mice. (a) Example of a section processed for BDNF immunohistochemistry across the hippocampal region of a P2 euploid mouse. This section encroaches the caudal cortex (cCX) and the caudal subventricular zone (cSVZ) of the current study. Calibration = 25 µm. (b, c) Optical density of BDNF immunoreactivity in the cSVZ (b) and in layer II of the cCX (c) of untreated and treated euploid and Ts65Dn mice. Data are given as fold difference vs. untreated euploid mice. The roman numerals indicate the cortical layers. Abbreviations: CA1, hippocampal field; IZ, intermediate zone; LV, lateral ventricle; SP, subplate; SVZ, subventricular zone.

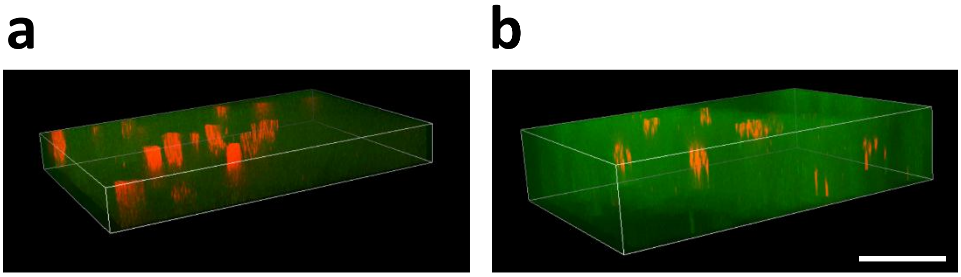

Suppl. Fig. 2. Penetration of the anti-BrdU antibody. 3-D reconstruction (Z-stack = 1µm) of BrdU-positive cells in the depth of the granule cell layer of the dentate gyrus of a P2 (a) and an adult (b) mouse. Sections were subjected to fluorescence immunohistochemistry for BrdU (red) and NeuN (green). Images were taken with an oil immersion 100x objective (N A 1.45; Nikon Plan Apo λ). 3-D reconstruction was carried out using NIS-Elements AR 4.30.02. Note that BrdU-positive cells can be detected close to the lower border of the section, indicating complete penetration of the antibody. Calibration = 30 µm.

SUPPLEMENTARY TABLES

Supplementary Table 1. Number of serial sections used in P2 mice for the evaluation of proliferation potency in each region of interest.

ROI	rSVZ	cSVZ	DG	rCX	cCX	STR	TH	HYP
Eu+Veh	7-8	7-10	6-9	6-7	7-8	6-7	6-7	6-8
Ts+Veh	5-6	6-7	5-8	5-6	6-7	4-8	5-6	6-7
Eu+7.8-DHF	6-8	7-9	6-8	6-7	6-9	6-7	5-7	6-8
Ts+7,8-DHF	5-7	6-10	7-9	5-7	6-9	6-8	5-8	6-8

The numbers in each column indicate the number of serial sections across each region of interest used for evaluation of the number of BrdU-positive cells. These sections encompassed the whole region of interest. Abbreviations: 7,8-DHF, 7,8-dihydroxyflavone: cCX, caudal cortex; cSVZ, caudal subventricular zone; DG, dentate gyrus; Eu, euploid; HYP, hypothalamus; rCX, rostral cortex; rSVZ, rostral subventricular zone; ROI, region of interest; STR, striatum; TH, thalamus; Ts, Ts65Dn; Veh, Vehicle.

Supplementary Table 2. Number of P2 mice used for the evaluation of proliferation potency and stereology in the examined regions.

BrdU immunohistochemistry

ROI	rSVZ	cSVZ	DG	rCX	cCX	STR	TH	HYP
ME	Tot	Tot	Tot	Tot	Tot	Tot	Tot	Tot
Eu+Veh	8	9	9	9	9	9	9	9
Ts+Veh	7	7	6	7	6	7	7	7
Eu+7,8-DHF	7	8	6	8	7	8	8	8
Ts-7,8-DHF	9	10	11	11	11	10	10	10

Nissl Staining

ROI	cCX L II	cCX L VI	cCX	DG	DG	DG	CA1	CA1	CA1
ME	De	De	Thick	Vo	De	Tot	Vo	De	Tot
Eu+Veh	7	7	7	6	6	6	6	6	6
Ts+Veh	6	7	8	6	5	5	5	5	5
Eu+7,8-DHF	5	5	5	5	5	5	5	5	5
Ts-7,8-DHF	6	8	8	8	8	8	8	8	8

The numbers in each column indicate the number of mice used for the measurements (ME), indicated in the corresponding row, in the different regions of interest (ROI), indicated in the corresponding row. Sections processed for BrdU immunohistochemistry were used to evaluate the total number of proliferating cells. Nissl-stained sections were used for stereology. Abbreviations: 7,8-DHF, 7,8-dihydroxyflavone: CA1, hippocampal field; cCX, caudal cortex; cSVZ, caudal subventricular zone; De, cell density; DG, dentate gyrus; Eu, euploid; HYP, hypothalamus; L II, layer two; L VI, layer sixth; rCX, rostral cortex; rSVZ, rostral subventricular zone; STR, striatum; Thick, thickness; TH, thalamus; Tot, total cell number; Veh, Vehicle; Vo, volume.
